# Potential landscape of high dimensional nonlinear stochastic dynamics with large noise

**DOI:** 10.1038/s41598-017-15889-2

**Published:** 2017-11-17

**Authors:** Ying Tang, Ruoshi Yuan, Gaowei Wang, Xiaomei Zhu, Ping Ao

**Affiliations:** 1 Department of Physics and Astronomy, Shanghai Jiao Tong University, Shanghai, 200240 China; 2 School of Biomedical Engineering, Shanghai Jiao Tong University, Shanghai, 200240 China; 3 Key Laboratory of Systems Biomedicine Ministry of Education, Shanghai Center for Systems Biomedicine, Shanghai Jiao Tong University, Shanghai, 200240 China; 4 Present Address: Department of Systems Biology, Harvard Medical School, 200 Longwood Avenue, Boston, Massachusetts, 02115 USA

**Keywords:** Nonlinear dynamics, Stochastic modelling

## Abstract

Quantifying stochastic processes is essential to understand many natural phenomena, particularly in biology, including the cell-fate decision in developmental processes as well as the genesis and progression of cancers. While various attempts have been made to construct potential landscape in high dimensional systems and to estimate transition rates, they are practically limited to the cases where either noise is small or detailed balance condition holds. A general and practical approach to investigate real-world nonequilibrium systems, which are typically high-dimensional and subject to large multiplicative noise and the breakdown of detailed balance, remains elusive. Here, we formulate a computational framework that can directly compute the relative probabilities between locally stable states of such systems based on a least action method, without the necessity of simulating the steady-state distribution. The method can be applied to systems with arbitrary noise intensities through A-type stochastic integration, which preserves the dynamical structure of the deterministic counterpart dynamics. We demonstrate our approach in a numerically accurate manner through solvable examples. We further apply the method to investigate the role of noise on tumor heterogeneity in a 38-dimensional network model for prostate cancer, and provide a new strategy on controlling cell populations by manipulating noise strength.

## Introduction

Studying stochastic dynamics is a central task to understand various natural and experimental phenomena in physics^1,2^, chemistry^3^, and biology^4–6^. Specifically, stochastic transitions induce current switching in a semiconductor^7^, reveal population stabilization^8^ or extinction^9^, and provide a mechanistic understanding for the genesis and progression of complex diseases such as cancers^10,11^. Potential landscape^12–14^, emergent from the underlying dynamical system, serves as a powerful tool to quantify multi-stability and estimate transition rates. However, a general approach to obtain this potential landscape in practice remains elusive. A major challenge is that real-world systems are intrinsically high dimensional, e.g. the gene regulatory network^4,10^, which makes simulation based approaches^15^ computationally unfeasible. In addition, systems may also subject to significant random fluctuations^16–18^ that have functional roles such as driving cell fate decisions^5,19–21^. To properly integrate such noise effects can be challenging as well.

To be specific, previous attempts, which compute the potential landscape as the logarithm of a simulated steady-state distribution, suffer from the exponentially increasing computational cost of stochastic simulations, and thus encounter the curse of dimensionality. More efficient simulation approaches are developed when the detailed balance condition is satisfied^22^, however, this condition breaks down for nonequilibrium systems^23^. Except simulations, methods on the basis of the Wentzel-Kramers-Brillouin (WKB) approximation^24^ or Freidlin-Wentzell theory^25–28^ are proposed, but their applications are restricted to systems with small noise. Therefore, on one hand, the high dimensionality defies the use of the expensive stochastic simulation; on the other hand, stochastic simulation seems inevitable except when the noise of the system is small. This conundrum avoids quantifying stability and stochastic transitions in real-world systems.

Towards resolving this problem, we notice that information critical for many applications can be extracted from the stable states, their relative steady-state probabilities and stability. These stable states have direct correspondence to experimental observables such as the biological phenotypes^21,29,30^. To obtain such information, we develop a computational framework based on the path integral, through a least action principle and the A-type interpretation^31,32^ of the stochastic differential equation (SDE). A dual role potential function lies at the core of the A-type interpretation, which exactly corresponds to 1) the steady-state distribution for the SDE and 2) the Lyapunov function^33^ for the SDE’s deterministic counterpart, an ordinary differential equation (ODE). With this correspondence, the relative probability between stable states can be computed directly, without the necessity of simulating the whole steady-state distribution. As a result, the present method has two major advantages: 1) it greatly reduces the computational cost as we focus on calculating the potential difference between stable states; 2) it is robust under arbitrary noise strength through the general consistency between ODE and SDE, and thus break the small noise restriction.

Our approach can be applied to a wide range of high dimensional stochastic dynamics, and enables us to investigate the role of large noise. It provides an efficient way to calculate probability ratios and transition rates between stable states. We demonstrate the method in a numerically accurate manner through an example with various noise intensities, and then apply it to a 38-dimensional network model for prostate cancer^11^. In particular, the tumor heterogeneity^6,29,34^ is observed controllable by the noise intensity. The result may uncover a mechanistic basis for hyperthermia.

## Results

### Formulation

#### Deviation between ODE and SDE

**Figure 1:**
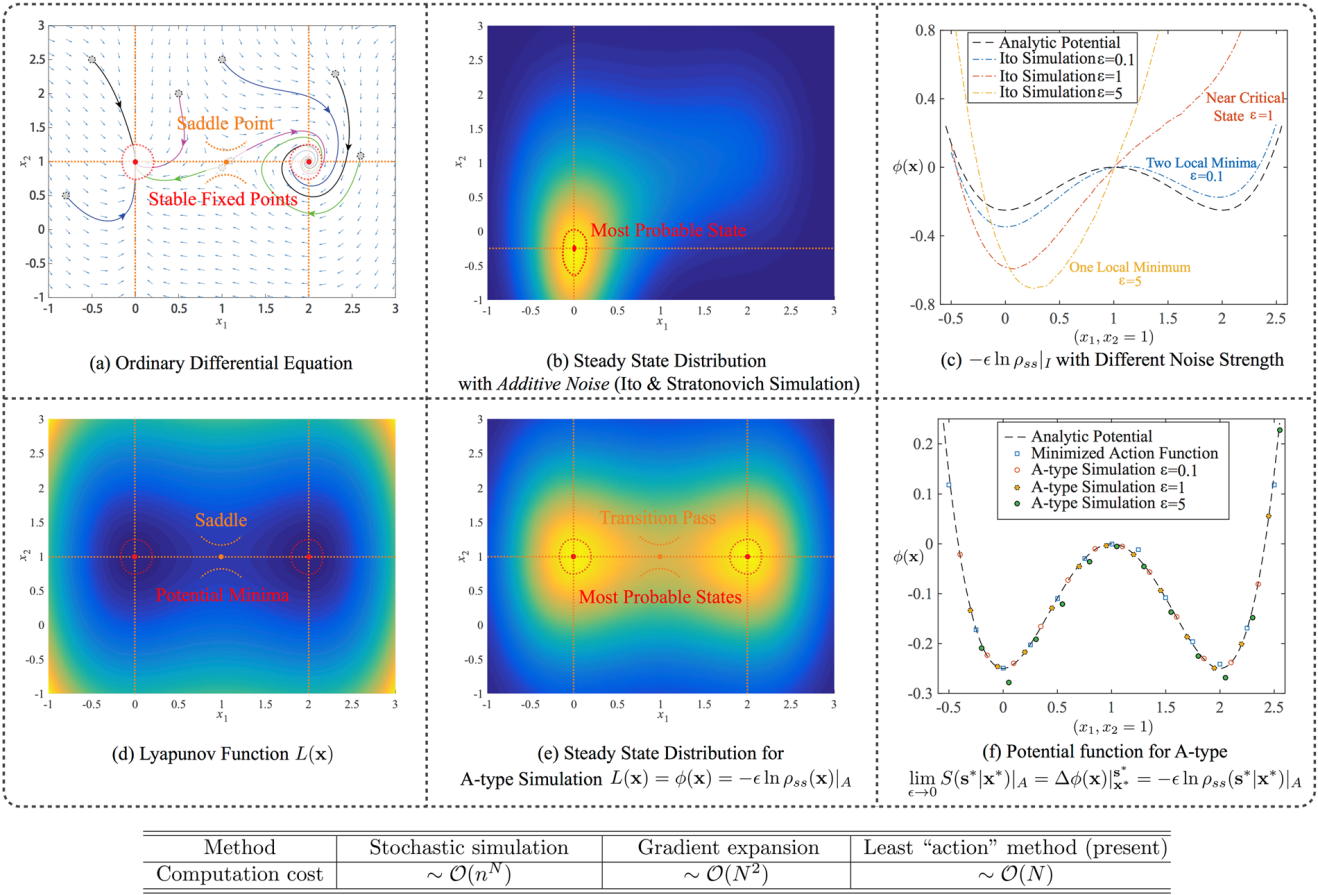
The modeling results on the potential (Lyapunov) function and steady-state distribution for an illustrative example with additive noise. Top panel: the prevailing stochastic simulation leads to a deviated stability structure. Bottom panel: The Lyapunov function, A-type simulation and potential function by least action method consistently reveal stability structure. (**a**) Vector field showing two stable fixed points and one saddle point. (**b**) Steady-state distribution by Ito’s (Stratonovich’s) simulation of SDE with *ε* = 1, where positions of fixed points from ODE and bistable topology are altered by large noise. (**c**) Deviations on potential values along the line *x*_2_ = 1 between the analytical construction and Ito’s simulation of SDE with noise strengths *ε* = 0.1, 1, 5. (**d**) Lyapunov function for ODE. (**e**) Steady-state distribution by A-type simulation of SDE with *ε* = 1, where positions of fixed points and bistable topology are consistent with those from ODE and Lyapunov function. (**f**) Potential values along the line *x*_2_ = 1 calculated by least action method, and A-type simulation of SDE with noise strengths *ε* = 0.1, 1, 5. Parameters are: *d* = *a* = *H* = 1. Table: computational costs of the methods on calculating potential barrier between fixed points with respect to system’s dimension *N*, where *n* is the number of mesh points in each dimension for stochastic simulation.

ODE and SDE can model a wide range of dynamics^33,35^, for example, chemical reactions^2^, population stabilization^8^, and carcinogenesis^11,30^. ODEs have been successfully used to quantitatively model average behaviors of a stochastic process^10,36–38^, for example, stable states correspond to biological phenotypes^39^; SDEs, by further adding a vanishing-mean noise term, recapitulate the stochastic process^5,19,40,41^. A major advantage of SDE modeling is the extractable dynamical information from its ODE counterpart, which may greatly reduce the computational cost of stochastic modeling. However, positions of the fixed points from the ODE can be altered after adding noise, under standard Ito’s or Stratonovich’s^35^ interpretation, known as the “noise effects”^42–45^. We also demonstrate here that even for a two dimensional example with simple additive white noise, the dynamical structure can be greatly altered by noise, e.g. from multi-stable to uni-stable, when applying prevailing simulation methods like Ito’s or Stratonovich’s^35^, as shown in Fig. 1(a–c). Such deviation is expected to be dramatic when modeling real-world systems with significant noises^16–18^. The methods based on WKB approximation^24^ or Freidlin-Wentzell theory^25–28^ encounter this problem, and thus they are mainly applied to the cases with small noise.

We identify two independent causes for the deviation between ODE and SDE when using the prevailing stochastic integrations: 1) the existence of a non-detailed balance part; 2) a variable-dependent diffusion matrix (multiplicative noise). Both causes are related to the freedom of choosing a stochastic interpretation for SDE. With the presence of multiplicative noise, it is known that conventional stochastic integrations like Ito’s and Stratonovich’s lead to distinct modeling results^35^. However, the deviation by non-detailed balance has not been noticed before, and it can occur even for systems with additive noise, where Ito’s and Stratonovich’s show no difference. Therefore, the prevailing stochastic simulations could not achieve the consistency for systems without detailed balance. More details are given in Sect. I of Supplemental Information.

Regarding the issues raised above, one may ask the following question: Is there a possibility to eliminate such unexpected “noise effects” and establish a general consistency between ODE and SDE modeling even under large fluctuations? If possible, dynamical information from the ODE counterpart can be inherited by the SDE modeling, such that the valleys’ positions in the landscape of SDE is obtainable by calculating stable states of ODE. The consistency is particularly necessary in a scenario where an ODE model is properly constructed and quantitatively correspond to experimental data on average, but are invalidated by the usual way of simulating SDE in reconstituting the original stochastic process and vice versa.

#### Bridging ODE and SDE

We provide a background on the framework that bridges ODE and SDE:

1 $$\dot{{\bf{x}}}={\bf{f}}({\bf{x}}),$$

2 $$\dot{{\bf{x}}}={\bf{f}}({\bf{x}})+G({\bf{x}})\zeta (t),$$

where the *N*-dimensional vector **x** denotes state variables, and $\dot{{\bf{x}}}$ represents its time evolution. The deterministic part is **f**(**x**), and the *M*-dimensional Gaussian white noise *ζ*(*t*) has 〈*ζ*(*t*)〉 = 0, 〈*ζ*(*t*)*ζ*^*τ*^(*t*′)〉 = 2*εδ*(*t* − *t*′), where *ε* is the noise strength playing the role of temperature, the superscript *τ* denotes transpose, *δ*(*t* − *t*′) is the Dirac delta function, and $\langle \cdots \rangle$ represents noise average. Here, *G*(**x**)*G*^*τ*^(**x**) = *D*(**x**) defines the symmetric positive definite diffusion matrix *D*(**x**). The multiplicative noise *G*(**x**)*ζ*(*t*) models that system state can in turn regulate noise by feedback, or inhomogeneity of the noisy environment. The results below are valid for multiplicative noise. For Eq. (2) with multiplicative noise, a freedom in choosing the integration method leads to different stochastic interpretations^35^, and we will specify a choice below.

It is challenging to generally construct Lyapunov function^33^ for ODEs, because **f** is typically nonlinear and cannot be written directly as the gradient of a potential function *U*(**x**): ${\bf{f}}\ne -\,{\nabla }_{{\bf{x}}}U$. Even so, a decomposed dynamics equivalent to Eq. (2) was discovered^14^, and Eqs (1) and (2) can be coherently decomposed as:

3 $${\bf{f}}({\bf{x}})=-\,[D({\bf{x}})+Q({\bf{x}})]\nabla L({\bf{x}}),$$

4 $$\dot{{\bf{x}}}=-\,[D({\bf{x}})+Q({\bf{x}})]\nabla \varphi ({\bf{x}})+G({\bf{x}})\,\ast \,\zeta (t),$$

where the matrix *Q*(**x**) is anti-symmetric with $\nabla {\varphi }^{\tau }Q\nabla \varphi =0$, and the asterisk means A-type stochastic integration^31^. A Lyapunov function *L*(**x**) and a potential function *ϕ*(**x**) are constructed, and *L*(**x**) satisfies *dL*/*dt* ≤ 0 for any trajectory of Eq. (1) ^46–48^. For Eq. (4), by solving the corresponding Fokker-Planck equation (FPE):

5 $${\partial }_{t}\rho ({\bf{x}},t)={\nabla }_{{\bf{x}}}^{\tau }[D({\bf{x}})+Q({\bf{x}})][{\nabla }_{{\bf{x}}}\varphi ({\bf{x}})+\varepsilon {\nabla }_{{\bf{x}}}]\rho ({\bf{x}},t),$$

which is obtained from the zero-mass limit on a 2*N*-dimensional Klein-Kramers equation^32^, the steady state obeys Boltzmann-Gibbs distribution ${\rho }_{ss}({\bf{x}})=\exp [\,-\,\varphi ({\bf{x}})/\varepsilon ]$. As the steady state is invariant under transformation *ϕ* → *ϕ* + *C* for any constant *C*, we have chosen *C* such that the distribution is normalized.

*A*-*type integration* is defined as the connection between the SDE (2) and the FPE (5), and realized by two explicit limiting procedures: first the usual integration limit and then the zero mass limit^32^. Even for systems with additive noise, it is different from the conventional *α*-type stochastic integration^31^ (*α* = 0 is Ito’s, *α* = 1/2 is Stratonovich’s), except that when detailed balance condition holds (*Q* = 0) it corresponds to *α* = 1. An exact transformation from A-type integration to Ito’s has been achieved^31^. *A*-*type simulation* can thus be implemented as follows: Eq. (4) is transformed to be an equivalent SDE under Ito’s interpretation:

6 $$\dot{{\bf{x}}}={\bf{f}}({\bf{x}})+\varepsilon {\rm{\Delta }}{\bf{f}}({\bf{x}})+G({\bf{x}})\cdot \zeta (t),$$

where ${\rm{\Delta }}{{\bf{f}}}_{i}({\bf{x}})={\sum }_{j}\,{\partial }_{{x}_{j}}[{D}_{ij}({\bf{x}})+{Q}_{ij}({\bf{x}})]$, and the dot denotes Ito’s integration. Thus, one can simulate Eq. (6) with Ito’s scheme to realize A-type simulation for Eq. (2).

An advantage led by the A-type simulation is that for arbitrary noise strength the sampled steady-state distribution of Eq. (2) corresponds to the Lyapunov function and potential function:

7 $$L({\bf{x}})=\varphi ({\bf{x}})={-\varepsilon \mathrm{ln}{\rho }_{ss}({\bf{x}})|}_{A},$$

where the subscript A denotes A-type simulation.

Then, the deterministic part of Eq. (2) has the same decomposition as Eq. (1), and positions of fixed points from Eq. (1) are not changed after added noise. For Ito’s or Stratonovich’s integrations one cannot recognize fixed points of ODEs from the simulated distribution even for additive noise, as shown in Fig. 1(a–c). A-type simulation also reserves topology of the landscape for arbitrary noise strength^31,47^, as exemplified in Fig. 1(d–f). Note that two numerical methods have been used to estimate the steady-state distribution or potential function in Eq. 7: the A-type simulation^31^ and the gradient expansion method^14^. To implement these two methods, the matrix *Q*(**x**) needs to be solved.

### Efficient computation of potential difference

The essential information for multi-stable systems is the relative stability between stable states, which can be extracted from the potential difference^1,2^. Based on the property that fixed points for ODE and locally most probable states for SDE are identical in our framework, we have the following protocol for a typical situation, where two stable fixed points ${{\bf{x}}}_{1}^{\ast }$ and ${{\bf{x}}}_{2}^{\ast }$ are connected by a saddle point s* (Protocol I):

1. Calculate the potential difference between each stable fixed point and the saddle point ${{\rm{\Delta }}\varphi ({\bf{x}})|}_{{{\bf{x}}}_{1}^{\ast }}^{{{\bf{s}}}^{\ast }}$, ${{\rm{\Delta }}\varphi ({\bf{x}})|}_{{{\bf{x}}}_{2}^{\ast }}^{{{\bf{s}}}^{\ast }}$ by the least action method Eq. (14) below. The detail of finding the least action path is given in Methods. The potential difference between ${{\bf{x}}}_{1}^{\ast }$ and ${{\bf{x}}}_{2}^{\ast }$ is ${{\rm{\Delta }}\varphi ({\bf{x}})|}_{{{\bf{x}}}_{1}^{\ast }}^{{{\bf{x}}}_{2}^{\ast }}={{\rm{\Delta }}\varphi ({\bf{x}})|}_{{{\bf{x}}}_{1}^{\ast }}^{{{\bf{s}}}^{\ast }}-{{\rm{\Delta }}\varphi ({\bf{x}})|}_{{{\bf{x}}}_{2}^{\ast }}^{{{\bf{s}}}^{\ast }}$.

The path integral formulation for Eq. (2) is applied here to calculate the potential difference. The formulation needs to be consistent with the stochastic integration used^49^. For A-type integration, $P({{\bf{s}}}^{\ast },{T}_{2}|{{\bf{x}}}^{\ast },{T}_{1})={\int }_{{{\bf{x}}}^{\ast }}^{{{\bf{s}}}^{\ast }}\,{{\mathscr{D}}}_{A}\,{\bf{x}}\,\exp \{{-{S}_{T}[{\bf{x}}]|}_{A}\,/\varepsilon \}$, where the action ${{S}_{T}[{\bf{x}}]|}_{A}$ is a function of paths [**x**(*t*)] with x* and s* as start point at time *T*_1_ and end point at *T*_2_ separately. As we have transformed Eqs (2) to (6), it is more convenient to use the equivalent path integral formulation:

8 $$P({{\bf{s}}}^{\ast },{T}_{2}|{{\bf{x}}}^{\ast },{T}_{1})={\int }_{{{\bf{x}}}^{\ast }}^{{{\bf{s}}}^{\ast }}\,{{\mathscr{D}}}_{I}\,{\bf{x}}\,\exp \{{-{S}_{T}[{\bf{x}}]|}_{I}/\varepsilon \},$$

where the subscript I means Ito’s integration. The measure on paths is defined as: $${\int }_{{{\bf{x}}}^{\ast }}^{{{\bf{s}}}^{\ast }}\,{{\mathscr{D}}}_{I}{\bf{x}}\equiv {\mathrm{lim}}_{K\to \infty }\,{\prod }_{k=1}^{K-1}$$$$\int \,d{{\bf{x}}}^{k}/\sqrt{{\rm{\det }}\mathrm{[2}\pi \varepsilon dtD({{\bf{x}}}^{k-1})]}$$, where functions of **x** take pre-points in each interval. The time is discretized into *K* segments with $${T}_{1}={t}_{1} < \cdots  < {t}_{k} < \cdots  < {t}_{K}={T}_{2}$$, each interval being *dt* and **x**^*k*^ = **x**(*t*_*k*_). The action function:

9 $${{S}_{T}[{\bf{x}}]|}_{A}={\frac{1}{4}{\int }_{{T}_{1}}^{{T}_{2}}|}_{I}dt{[\dot{{\bf{x}}}-{\bf{f}}({\bf{x}})-\varepsilon {\rm{\Delta }}{\bf{f}}({\bf{x}})]}^{\tau }{D}^{-1}({\bf{x}})\,[\dot{{\bf{x}}}-{\bf{f}}({\bf{x}})-\varepsilon {\rm{\Delta }}{\bf{f}}({\bf{x}})],$$

where the integration obeys Ito’s rule^35^. Note that the present action function is different from that of Freidlin-Wentzell’s framework^25^ except when *ε* → 0. Even for SDE with additive noise, the difference still exists, because ${\rm{\Delta }}{{\bf{f}}}_{i}({\bf{x}})={\sum }_{j}\,{\partial }_{{x}_{j}}{Q}_{ij}({\bf{x}})$ can be nonzero for systems without detailed balance.

By using the decomposition in Eq. (4), we have:

10 $$\begin{array}{rcl}{{S}_{T}[{\bf{x}}]|}_{A} & = & {\frac{1}{4}{\int }_{{T}_{1}}^{{T}_{2}}|}_{I}dt{(\dot{{\bf{x}}}-D\nabla \varphi +Q\nabla \varphi -\varepsilon {\rm{\Delta }}{\bf{f}})}^{\tau }{D}^{-1}(\dot{{\bf{x}}}-D\nabla \varphi +Q\nabla \varphi -\varepsilon {\rm{\Delta }}{\bf{f}})\\  &  & +{{\int }_{{T}_{1}}^{{T}_{2}}|}_{I}dt{\dot{{\bf{x}}}}^{\tau }\nabla \varphi +{{\int }_{{T}_{1}}^{{T}_{2}}|}_{I}dt{(\nabla \varphi )}^{\tau }(Q\nabla \varphi -\varepsilon {\rm{\Delta }}{\bf{f}})\\  &  & {\ge \,{\rm{\Delta }}\varphi |}_{{{\bf{x}}}^{\ast }}^{{{\bf{s}}}^{\ast }}-\varepsilon \,{\int }_{{T}_{1}}^{{T}_{2}}\,dtD{\nabla }^{2}\varphi -{\varepsilon {\int }_{{T}_{1}}^{{T}_{2}}|}_{I}dt{(\nabla \varphi )}^{\tau }{\rm{\Delta }}{\bf{f}},\end{array}$$

where we have used $\nabla {\varphi }^{\tau }Q\nabla \varphi =0$, and Ito’s formula^35^: ${{\int }_{{T}_{1}}^{{T}_{2}}|}_{I}dt{\dot{{\bf{x}}}}^{\tau }\nabla \varphi ={{\rm{\Delta }}\varphi |}_{{{\bf{x}}}^{\ast }}^{{{\bf{s}}}^{\ast }}-\varepsilon \,{\int }_{{T}_{1}}^{{T}_{2}}\,dt\,D{\nabla }^{2}\varphi$ with $D{\nabla }^{2}$ denoting ${D}_{ij}{\partial }_{{x}_{i}}{\partial }_{{x}_{j}}$. For clarity, we ignore the symbol (**x**) for functions of **x** in the derivation. The inequality in Eq. (10) becomes equality for the least action path:

11 $$\dot{{\bf{x}}}=D({\bf{x}})\nabla \varphi ({\bf{x}})-Q({\bf{x}})\nabla \varphi ({\bf{x}})+\varepsilon {\rm{\Delta }}{\bf{f}}({\bf{x}}),$$

where the trajectory tends to go reversely from s* to x*. Thus, Eq. (9) counts the accumulation of noise, whose minimization equals to the “uphill” energy ${{\rm{\Delta }}\varphi ({\bf{x}})|}_{{{\bf{x}}}^{\ast }}^{{{\bf{s}}}^{\ast }}$. When the trajectory passes the saddle point, it goes “downhill” obeying $\dot{{\bf{x}}}=-\,D({\bf{x}})\nabla \varphi ({\bf{x}})-Q({\bf{x}})\nabla \varphi ({\bf{x}})+\varepsilon {\rm{\Delta }}{\bf{f}}({\bf{x}})$, where minimization of Eq. (9) is zero.

In the limit of *ε* → 0,

12 $${{S}_{T}[{\bf{x}}]|}_{A}\ge {{\rm{\Delta }}\varphi ({\bf{x}})|}_{{{\bf{x}}}^{\ast }}^{{{\bf{s}}}^{\ast }},$$

and the least action path follows the time-reversal adjoint dynamics^50^ of Eq. (1) with decomposition Eq. (3): $\dot{{\bf{x}}}=D({\bf{x}})\nabla \varphi ({\bf{x}})-Q({\bf{x}})\nabla \varphi ({\bf{x}})$. Since the dynamics is deterministic, the least action path connecting s* to x* is specified. Then, minimization of the action function

13 $${S({{\bf{s}}}^{\ast }|{{\bf{x}}}^{\ast })|}_{A}\doteq {\mathop{{\rm{\inf }}}\limits_{\{T > 0\}}\mathop{{\rm{\inf }}}\limits_{\{x({T}_{1})={x}^{\ast },x({T}_{2})={s}^{\ast }\}}{S}_{T}[{\bf{x}}]|}_{A}$$

equals to the potential difference between s* and x*, which is unique if the ideal least action path following $\dot{{\bf{x}}}=D({\bf{x}})\nabla \varphi ({\bf{x}})-Q({\bf{x}})\nabla \varphi ({\bf{x}})$ is found.

With Eq. (7), we reach a formula to calculate potential barrier:

14 $${\mathop{\mathrm{lim}}\limits_{\varepsilon \to 0}S({{\bf{s}}}^{\ast }|{{\bf{x}}}^{\ast })|}_{A}={{\rm{\Delta }}\varphi ({\bf{x}})|}_{{{\bf{x}}}^{\ast }}^{{{\bf{s}}}^{\ast }}={-\varepsilon \mathrm{ln}{\rho }_{ss}({{\bf{s}}}^{\ast }|{{\bf{x}}}^{\ast })|}_{A}\mathrm{.}$$

We emphasize that the first equality holds only when *ε* → 0, but the second equality is for arbitrary noise strength, which is different from that of Freidlin-Wentzell’s framework valid for *ε* → 0^25^. The significance of Eq. (14) is that it enables to obtain potential difference by minimizing the action when *ε* → 0, and the result exactly corresponds to that of Lyapunov function for ODE and by A-type simulation of SDE with arbitrary noise strength.

### Probability ratio and transition rate

As the steady state obeys the Boltzmann-Gibbs distribution, the probability ratio between stable states is:

15 $$\frac{\rho ({{\bf{x}}}_{2}^{\ast })}{\rho ({{\bf{x}}}_{1}^{\ast })}=\exp \,[{-\frac{1}{\varepsilon }{\rm{\Delta }}\varphi ({\bf{x}})|}_{{{\bf{x}}}_{1}^{\ast }}^{{{\bf{x}}}_{2}^{\ast }}],$$

cell state where ${{\rm{\Delta }}\varphi ({\bf{x}})|}_{{{\bf{x}}}_{1}^{\ast }}^{{{\bf{x}}}_{2}^{\ast }}={{\rm{\Delta }}\varphi ({\bf{x}})|}_{{{\bf{x}}}_{1}^{\ast }}^{{{\bf{s}}}^{\ast }}-{{\rm{\Delta }}\varphi ({\bf{x}})|}_{{{\bf{x}}}_{2}^{\ast }}^{{{\bf{s}}}^{\ast }}$. Equation (15) is valid under arbitrary noise strength, and thus can show variation of the ratio with different noise intensities. It provides the probability ratios of quantities such as the number of different cell states. Specifically, we apply it to analyze tumor heterogeneity by large noise in the section of Application.

When *ε* is small compared to the height of the potential barrier ${{\rm{\Delta }}\varphi ({\bf{x}})|}_{{{\bf{x}}}_{1}^{\ast }}^{{{\bf{s}}}^{\ast }}$, the asymptotic transition rate formula^1,2^ from the stable fixed point ${{\bf{x}}}_{1}^{\ast }$ to ${{\bf{x}}}_{2}^{\ast }$ is:

16 $$R({{\bf{x}}}_{2}^{\ast }|{{\bf{x}}}_{1}^{\ast })\propto \exp \,[{-\frac{1}{\varepsilon }{\rm{\Delta }}\varphi ({\bf{x}})|}_{{{\bf{x}}}_{1}^{\ast }}^{{{\bf{s}}}^{\ast }}].$$

Different from^51^, in our framework the non-detailed balance part does not provide correction terms that explicitly appear in the pre-factor of the rate formula as analyzed in Sect. IV of Supplemental Information.

### Comparison on computational cost

Computational costs of methods mentioned above for systems with respect to dimension *N* are analyzed here. For stochastic simulation of Eq. (4), we mesh each dimension into *n* points, and the computational cost is exponentially proportional to dimension, $cost\sim {\mathscr{O}}({n}^{N})$. This method also has the problem of slow convergence when noise strength is small. For the gradient expansion^14^, as in each step a matrix needs to be evaluated, the computational cost is approximately $cost\sim {\mathscr{O}}({N}^{2})$. Both of the two methods need to solve the matrix *Q* for A-type interpretation.

However, information critical for many stochastic models is mainly obtained from estimating the relative probabilities between stable states. Under such circumstances, it is sufficient to find the potential differences between fixed points, without the necessity of obtaining the whole steady-state distribution. Thus, we only need to calculate the one dimensional least action path connecting fixed points, and the computational cost should be linearly proportional to the dimension, $cost\sim {\mathscr{O}}(N)$. As a result, the least action method is efficient in high dimensional systems. We list the results in the table of Fig. 1.

### Protocol to obtain a global landscape

Protocol I can be extended to obtain a global landscape for systems with multiple stable states (Protocol II):

1. Find the fixed points under consideration from solving the ODE counterpart, such as by Newton iteration method^52^.
2. Classify all the fixed points into two groups by calculating the eigenvalues of the linearized Jacobian matrix in their neighborhood: stable fixed points (no eigenvalue with positive real part) and unstable points (at least one eigenvalue with positive real part).
3. Choose a saddle point as reference. Start from the points in a small neighborhood of the saddle point, and simulate the ODE to find all the stable fixed points reached. Calculate potential difference between the saddle point and the stable fixed points by the least action method in Eq. (14). The detail of the minimization procedure in Eq. (14) is given in Methods.
4. Repeat step 3 for all saddle points. Assign relative potential difference between the saddle points if they reach a common stable fixed point.
5. For any other points in state space, simulate the ODE to find the fixed point it reaches. Obtain their potential difference by the least action method. The total computational cost depends on the potential value of how many points are calculated.
6. With the calculated potential values, extract the relative probabilities between the states by Eq. (15).

The consistency between ODE and SDE enables to utilize information of fixed points and basins of attraction from ODE, which greatly improves the efficiency of our algorithm. As each point in state space reaches a single fixed point, its potential value is uniquely determined if the ideal least action method is found numerically during minimization procedure, which leads to a global landscape without ambiguity. Specifically, the probability ratio between a fixed and a point within the potential well is calculated by Eq. (15), which corresponds to cell-to-cell variability inside the attractor. For dynamical systems with complex attractors such as limit cycle^33,47,53^, the point on the stable limit cycle can be treated similarly as the stable fixed point, and thus our method can be generalized.

### Application

Heterogeneity of cell populations is widely observed in biological systems such as cancer^54^, where different cell phenotypes emerge in tumor tissues^34,55^. It is proposed that an underlying regulation network and quantification on the network dynamics by SDE models can describe various cell states and transitions between them^6,15^. Starting from the SDE model, the calculated potential landscape provides an integrated picture to study heterogeneity. Specifically, valleys in the landscape correspond to different cell states, and the potential barrier separating them quantifies the transition rates. This approach of landscape is helpful to understand systematically the effect of perturbations on cell state interconversions.

Here, we investigate whether the variation of noise strength leads to changes of cell states, as noise plays a crucial role in biological processes, for example, it drives the cell fate decision^20,21^. The previous methods^24–28^ can not be applied to study the function of large noise, because identification on valleys of landscape and calculation on potential barrier by these methods are restricted to the zero noise limit. Now, we are able to quantify the role of large noise on heterogeneity in high dimensional network dynamics, because the present calculation on landscape is robust under arbitrary noise strength. From our method, the ratios of cell states can be controlled by manipulating noise strength, which allows the cell-to-cell variability under the same gene regulation network.

#### Prostate cancer model

As an illustrative example, we demonstrate the effectiveness of our approach by applying it to a network model for the prostate cancer^11^. The network dynamics is modeled by a 38-dimensional SDE. Each dynamical variable represents the expression or activity level of a gene. The dynamics of each component is written as a sum of the generation and degradation, including the activation or inhibition by the other genes. We use the standard Hill equation to model such interactions^56,57^.

**Figure 2:**
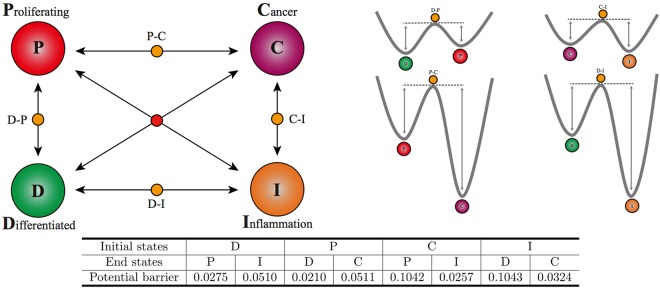
Potential barriers calculated by Protocol II in the prostate cancer model^11^. Left panel: The chosen four cell states: differentiated (D), proliferating (P), cancer (C), inflammation (I). They are stable fixed points obtained from ODE of the 38-dimensional system. The states D-P, D-I, C-I, P-C are saddle points, and the red fixed point in the middle is unstable. Right panel: the heights of potential barriers between stable fixed points connected by saddles. The lengths of arrows are proportional to barrier heights listed in the table below. Table: potential barriers between stable fixed points are calculated by the least action method. We set *K* = 100, *T* = 20, and have checked that larger *K* and *T* values lead to convergent results. The parameters of the system chosen here are for typical cancer patients^11^, where cancer and inflammation states are more stable.

The ODE counterpart and the parameters are given in Sect. VII of the Supplemental Information. From the analysis on the ODE, we know the system has 10 stable fixed points and 16 saddle points. We find that four stable fixed points correspond to various cell states shown in Fig. 2: differentiated (D), proliferating (P), cancer (C), inflammation (I) with their positions given in^11^. For clarity, we consider additive noise case with diffusion matrix *D*(**x**) as an identity matrix in this example. It should be emphasized that the deviation between ODE and SDE appears even with additive noise, because this system does not obey the detailed balance condition. As a result, considering additive noise is sufficient to demonstrate the advantage of our method based on A-type integration compared with the prevailing methods based on other stochastic integrations.

**Figure 3:**
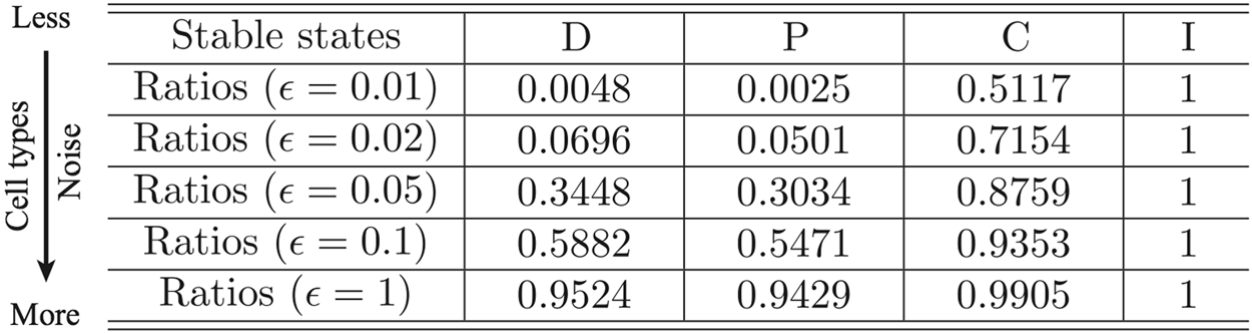
The probability ratios between D, P, C, I states of the prostate cancer model. The ratios are calculated by Eq. (15) with heights of potential barriers listed in the table of Fig. 2, where the values are normalized by the I state. We consider noise intensities: *ε* = 0.01, 0.02, 0.05, 0.1, 1. The tumor heterogeneity emerges when noise strength becomes large where the four types of cells are almost equally distributed.

We use the least action method to calculate heights of the potential barriers between stable states, as shown in Fig. 2. We note that in order to have the global landscape, we use the continuous condition to set the potential value of *P* to *D* specifically. According to Eq. (15), the probability ratios of different cell states can be calculated under various noise strengths. We list the ratios between the states of D, P, C, I in Fig. 3, which shows qualitative different results with changing noise. When noise is small, e.g. *ε* = 0.01, most of the cells belong to the cancer state and the inflammation state, whereas little are in the differentiated state and the proliferating state. When noise becomes large such as *ε* = 1, various cell states are almost equal in number. This demonstrates the emergence of tumor heterogeneity with respect to increasing the noise strength.

We elucidate more on the application of controlling the ratios of cell states through varying the noise intensity, which can be implemented by tuning temperature. First, it was demonstrated that an therapy with combination of hyperthermia and other treatments, such as immunotherapy and radiotherapy, can improve the efficiency of cancer cure^58^. In those cases, temperature plays the role of enhancer to switch on and off the effectiveness of other therapies. Specifically, drug cytotoxicity triggered by temperature variation leads to the death of tumor cells, and therefore combination of hyperthermia and chemotherapy is regarded as an effective treatment of cancer^59^. Second, as different levels of heating were found to bring distinct modulatory effect on tumor targets, our method valid for arbitrary noise strength may be applied to study sensitivity of thermal treatment regulated by temperature, which will provide new designs on clinical trials. Third, the regional hyperthermia to radiotherapy^60^ shows an improvement on survival rates of cancer patients, because hyperthermia can guide the action of chemotherapy to specific heated tumor region. This can be modeled by multiplicative noise, as exemplified in Sect. I of the Supplemental Information. Therefore, the present approach could form the theoretical basis for hyperthermia that employs effect of temperature in tumor treatment.

With the obtained potential barrier, transition rates of cell state interconversions are given by Eq. (16), which provides a quantitative understanding on the cancer genesis. Under the given parameters and noise strength, the result provides a set of predictions: 1) the cancer and the inflammation states are more stable than the proliferating and the differentiated states; 2) transitions from the cancer state to the proliferating state and from the inflammation state to the differentiated state are difficult than the other way around; 3) transition to the cancer state from the inflammation state is more frequent than from the proliferating state. These suggest that the model may describe a cancer patient, and new strategies for medical treatments should be designed to raise the potential energy of the cancer and the inflammation states.

## Discussion

The above results depend on a specific choice on the stochastic integration. From the mathematical aspect, different stochastic integrations are equivalent, and can be transformed to each other by modifying the drift term **f**(**x**) correspondingly. From the physical aspect, for a system with clearly separated sources for the deterministic force and the stochastic force, i.e. the two forces can be measured independently in an experiment, we might say that a stochastic interpretation for the SDE is chosen by Nature. There are particular scenarios in which such measurements are possible, for example, the experiments^61,62^ have shown that A-type is chosen for a class of systems, which demonstrates that the A-type integration is not only a theoretical treatment.

However, in many extended applications such as phenomenological models for biological systems, separating deterministic and stochastic forces would not even be meaningful, and we would rather consider stochastic integrations as mathematically equivalent tools that is chosen by modeling. Under the situation, SDE model set by a combination of the drift term **f**(**x**) and stochastic integration can be non-unique, and each is an effective description for the system. If the deterministic rate equations are properly constructed and quantitatively correspond to the experimental measurements on average, e.g. the stable fixed points from the ODE part are consistent with the distribution peaks from data, then A-type integration should be a proper choice for calculating the steady-state distribution. Because the correspondence between ODE and SDE modeling under arbitrary noise strength is a property for the A-type integration. The cancer model discussed above belongs to this category, as its ODE part was demonstrated to match the experimental observation on average^11^.

In biology, noise has a variety of sources^63^, such as locations of molecules, micro-environmental fluctuations, gene expression noise, and cellular processes like cell growth. For complex systems like cancer, noise may come from different sources. SDE model reconstitutes the random fluctuations into a single noise term, which reflects the various sources of noise^19,64^. Therefore, several experimental operations can implement the change of the noise strength discussed here in real biological systems.

Our method can be applied to systems that are modeled by master equation (CME) with discrete dynamical variable^35^. First, CME can be transformed to be the chemical Langevin equation with continuous variable^40^, which can be cast into the form of Eq. (2). Then, our method is applicable to improve efficiency. The approximation is tolerably accurate when the copy number of variables are large, and it also requires that the dynamical process has a time scale during which multiple reactions occur and the reaction rate does not change dramatically^40^. These conditions are expected to hold for the present high dimensional cancer dynamics^10,11^, where the proteins usually has high copy numbers. Second, CME may also be expanded to a FPE and further corresponds to Eq. (4) with consistent modeling predictions, as demonstrated through an explicit procedure in Sect. II of Supplemental Information. Third, for systems with low copy numbers, SDE can still provide an appropriate description on the effect of noises^19^. Fourth, for stochastic processes on the level of single molecules, such as gene burst process^65^, CME is a more proper approximation to capture the discrete nature of species^9^. Nevertheless, this kind of noise will diminish by accumulation of proteins with long lifetime^66^.

Mathematically, SDE and CME are two independent modeling methodologies, and are on an equal footing to describe the stochastic dynamics. Both CME and SDE are models with intrinsic discrepancy to the real dynamical process. From computational side, a whole set of CME to describe the stochastic dynamics in detail is typically high dimensional, and the Gillespie algorithm^67^ to simulate CME is time consuming. Thus, the present method handling SDE valid for arbitrary noise strength is practically useful to investigate high dimensional systems with large fluctuations, particularly when the ODE counterpart is properly constructed and quantitatively correspond to the average experimental data.

Several other remarks are in order. First, our calculation on the potential difference is applicable to systems both with and without detailed balance condition^14^, i.e. *Q* = 0 or not. Breakdown of detailed balance inducing a curl flux in the state space affects the least action path, and generally leads to ${S({{\bf{x}}}_{1}^{\ast }|{{\bf{x}}}_{2}^{\ast })|}_{A}\ne {S({{\bf{x}}}_{2}^{\ast }|{{\bf{x}}}_{1}^{\ast })|}_{A}$. For such cases, the least action path also differ from the deterministic saddle-node trajectories^25^. Second, there are many efficient numerical methods to calculate fixed points of ODEs in high dimension^52^, such as Newton iteration method. Third, the present action function has the dimension of energy, and the conventional action in classical physics has the dimension of energy multiplied by time. Fourth, positions for the locally most probable states in Eq. (3) is a subset of fixed points’ positions for Eq. (1), because $\nabla \varphi ({\bf{x}})=0$ is sufficient but not necessary to ${\bf{f}}({\bf{x}})=-\,[D({\bf{x}})+Q({\bf{x}})]\nabla \varphi ({\bf{x}})=0$. Fifth, the constructed potential function is also useful to extract thermodynamical free energy for non-equilibrium systems^68,69^.

We next compare our framework with the previous works. First, authors in^24^ used a WKB method and reached a Hamiltonian-Jacobi equation $\nabla \varphi {({\bf{x}})}^{\tau }D({\bf{x}})\nabla \varphi ({\bf{x}})+\nabla \varphi {({\bf{x}})}^{\tau }{\bf{f}}({\bf{x}})=0$ in the lowest order of *ε* → 0. However, computational cost of solving this partial differential equation increases exponentially. This Hamiltonian-Jacobi equation is valid for arbitrary orders of *ε* in our framework^32^. Second, our method is different from the previous path integral approach^15^, where their action function gives an effective potential rather than the exact potential *ϕ*(**x**) constructed consistently in Eqs (3) and (4). Third, the unexpected “noise effects” in using SDEs have been widely reported^42–44^, and whether the phenomena are produced by the physical effect of the zero-mean noise or by the intricacy of using various stochastic integrations^45,70^ (also see the example in Sect. II of Supplemental Information) is a question without a definite answer. These effects in general defy the use of dynamical information from the ODE counterpart, and our method provide a possibility to reserve useful results by ODE analysis for SDE with arbitrary noise strength.

We analyze the difference between our framework and Freidlin-Wentzell’s^25^, based on which the quasi-potential has been calculated in many systems recently^26–28^. First, the consistency between action function’s form and stochastic integration (classified in Table II of Supplemental Information) has not been considered in Freidlin-Wentzell’s framework. Only the present action function of Ito’s form is the same as the usual Freidlin-Wentzell’s action^25^. Second, our decomposition ${\bf{f}}({\bf{x}})=-\,[D({\bf{x}})+Q({\bf{x}})]\nabla \varphi ({\bf{x}})$ with $\nabla \varphi ({\bf{x}})Q({\bf{x}})\nabla \varphi ({\bf{x}})=0$ is generally different form the usual Freidlin-Wentzell form ${\bf{f}}({\bf{x}})=-\,\nabla U({\bf{x}})+l({\bf{x}})$ with $\nabla U({\bf{x}})\cdot l({\bf{x}})=0$, except when diffusion matrix *D*(**x**) is proportional to identity. For general *D*(**x**), the minimization of action function Eq. (17) does not directly equal to the function *U*(**x**) even in the limit of *ε* → 0. For example, when *D*(**x**) is a diagonal matrix with distinct constant elements, the action ${{S}_{T}[{\bf{x}}]|}_{I}\ge {\sum }_{i}\,{\int }_{{T}_{1}}^{{T}_{2}}\,dt({\dot{x}}_{i}{\partial }_{{x}_{i}}U/{D}_{ii}-{l}_{i}{\partial }_{{x}_{i}}U/{D}_{ii})\ne {{\rm{\Delta }}U({\bf{x}})|}_{{{\bf{x}}}^{\ast }}^{{{\bf{s}}}^{\ast }}$. On the other hand, if we apply the action without the diffusion matrix as in^26^, ${\hat{S}}_{T}({\bf{x}})={\sum }_{i}\,{\int }_{{T}_{1}}^{{T}_{2}}\,dt{({\dot{x}}_{i}-{f}_{i})}^{2}/2$, it does not include the effect of *D*(**x**). A more detailed comparison is given in Sect. V of Supplemental Information.

Recently, the existence of decomposition Eq. (4) and its equivalence to Freidlin-Wentzell’s quasi-potential in the zero noise limit is discussed^71^. Here, we provide a framework to directly extend the potential function to the cases with finite noise. Besides, the classical Freidlin-Wentzell’s quasi-potential is locally constructed around each stable attractors, and complicated methods to glue the locally constructed quasi-potentials are required^25^. In this paper, we find that a unique potential function can be obtained by finding the ideal least action path for the minimization in Eq. (13). Even *Q* in Eq. (4) generally may not be uniquely determined for a given Eq. (2)^71^, certain boundary conditions or physical constrains can be added to specify *Q* in order to guarantee a unique least action path for Eq. (13).

In order to be more efficient in finding the least action path as given by the time-reversal adjoint dynamics, here we provide a strategy of minimization called ODE-based-adaptive-time method. First, we simulate ODE, Eq. (1), to get a path from s^*^ (considering s^*^ is a saddle point, add a small perturbation to it as the starting point) to a corresponding stable fixed point x^*^. We record the total duration for the trajectory as ${T}_{{s}^{\ast }\to {x}^{\ast }}^{ODE}$. Since the least action path is time-reversal of the adjoint dynamics, its duration and length should be the same as that of the corresponding trajectory for the original ODE. Therefore, we choose ${T}_{2}-{T}_{1}={T}_{{s}^{\ast }\to {x}^{\ast }}^{ODE}$, and put the constrain $in{f}_{\{{T}_{2}-{T}_{1}={T}_{{S}^{\ast }\to {x}^{\ast }}^{ODE}\}}$ to the minimization in Eq. (13). Note that the constrain of trajectory time here could not guarantee to find the ideal least action path, however, it can reduce the sampling space of paths connecting x^*^ to s^*^. To ensure that the ideal least action path is picked, new types of minimization algorithm considering dynamics by $-Q({\bf{x}})\nabla \varphi ({\bf{x}})$ need to be developed.

To conclude, we have identified two independent causes for the deviation between the SDE and its ODE counterpart when using the prevailing stochastic integrations: the existence of a non-detailed balance component or a variable-dependent diffusion matrix (multiplicative noise). We have developed a new numerical approach to study multi-stability and stochastic transitions between stable states for the SDE modeling. We are able to compute efficiently probability ratios between stable states in high dimensional nonequilibrium systems subject to large noise, through a least action method under the A-type stochastic interpretation. The modeling results of a prostate cancer network reveal a new mechanism to control the ratios of cell states by manipulating the noise intensity. Our approach should also be practically useful to study the role of the noise in the dynamical modeling of many other real-world high dimensional stochastic processes.

## Methods

We have demonstrated in Sect. III of Supplemental Information that differences of action functions for Eq. (2) with various stochastic integrations can be neglected when *ε* → 0. Therefore, we can choose action with specific stochastic integration for the convenience of numerical calculations. Here, we adopt the action with Ito’s integration^49^:

17 $${{S}_{T}[{\bf{x}}]|}_{I}={\frac{1}{4}{\int }_{{T}_{1}}^{{T}_{2}}|}_{I}dt{[\dot{{\bf{x}}}-{\bf{f}}({\bf{x}})]}^{\tau }{D}^{-1}({\bf{x}})\,[\dot{{\bf{x}}}-{\bf{f}}({\bf{x}})].$$

When *ε* → 0, its minimization equals to the potential difference ${{\mathrm{lim}}_{\varepsilon \to 0}S({{\bf{s}}}^{\ast }|{{\bf{x}}}^{\ast })|}_{I}={{\mathrm{lim}}_{\varepsilon \to 0}S({{\bf{s}}}^{\ast }|{{\bf{x}}}^{\ast })|}_{A}={{\rm{\Delta }}\varphi ({\bf{x}})|}_{{{\bf{x}}}^{\ast }}^{{{\bf{s}}}^{\ast }}$ with the least action path satisfying $\dot{{\bf{x}}}=D\nabla \varphi -Q\nabla \varphi$, and deviation between the least action path and that by $\dot{{\bf{x}}}=-\,D\nabla \varphi -Q\nabla \varphi +\varepsilon {\rm{\Delta }}{\bf{f}}$ for Eq. (9) disappears as well.

Numerically, we use discretized form of Eq. (17) as the object function to minimize. The discretized scheme adopted corresponds to the stochastic integration^49^, such as pre-point scheme is needed for Ito’s interpretation. Thus, we minimize the discretized action:

18 $${{S}_{T}[{\bf{x}}]|}_{I}=\frac{1}{4}\,\sum _{k=1}^{K}\,{\rm{\Delta }}{t}_{k}\,{[\frac{{{\bf{x}}}^{k}-{{\bf{x}}}^{k-1}}{{\rm{\Delta }}{t}_{k}}-{\bf{f}}({{\bf{x}}}^{k-1})]}^{\tau }{D}^{-1}({{\bf{x}}}^{k-1})\,[\frac{{{\bf{x}}}^{k}-{{\bf{x}}}^{k-1}}{{\rm{\Delta }}{t}_{k}}-{\bf{f}}({{\bf{x}}}^{k-1})],$$

where we divide the time intervals to be ${T}_{1}={t}_{0} < \cdots  < {t}_{k} < \cdots  < {t}_{K}={T}_{2}$ with ${\rm{\Delta }}{t}_{k}={t}_{k}-{t}_{k-1}$. The trajectory **x**(*t*) is divided into *K* pieces with **x**^*k*^ = **x**(*t*_*k*_). Besides, Eq. (17) is explicitly independent of *ε*, and thus its numerical minimization has implicitly taken the limit *ε* → 0. We choose a straight line connecting the two points as initial path, and set *T* = −*T*_1_ = *T*_2_. We use the fmincon function with the interior-point algorithm in the toolbox of MATLAB to do minimization. A package of MATLAB code to implement our method is provided in supplementary material. The minimization procedure requires to calculate gradient of the action function for the optimization step, and each step has a cost linearly proportional to dimension. Thus, in numerical realization the computational cost may become superlinear to dimension, and is at most quadratic to dimension. Such increasing demand on computational power is also encountered for the gradient expansion method. Therefore, both the stochastic simulation and the gradient expansion method are more computationally expensive than the present method.

For Eq. (2), when $Q({\bf{x}})\nabla \varphi ({\bf{x}})$ is dominate compared to $D({\bf{x}})\nabla \varphi ({\bf{x}})$, the least action path rotates and is relatively long in length. Then, the number of points *K* is required to be large enough such that the minimization procedure can find out the long least action path. Besides, we find in the numerical experiment that if *T* is too large, the least action path may pass an additional saddle point (limit cycle) with higher potential energy before going through the expected saddle point. Special care is needed to choose suitable *K* and *T* in these cases, and the ODE-based-adaptive-time method proposed in Discussion is a candidate to improve the efficiency. There are also numerical methods to adjust the grid points on the least action path, such as the adaptive minimum action method^72^. They can be applied to optimize our current numerical code.

## Electronic supplementary material

Supplementary Material

Supplementary Computational Material

## References

[1] Kramers HA (1940). Brownian motion in a field of force and the diffusion model of chemical reactions. Physica.

[2] Hänggi P, Talkner P, Borkovec M (1990). Reaction-rate theory: fifty years after Kramers. Rev. Mod. Phys..

[3] Eyring H (1935). The activated complex in chemical reactions. J. Chem. Phys..

[4] McAdams HH, Arkin A (1997). Stochastic mechanisms in gene expression. Proc. Natl. Acad. Sci. USA.

[5] Zhu X, Yin L, Hood L, Ao P (2004). Calculating biological behaviors of epigenetic states in the phage *λ* life cycle. Funct. Integr. Genomics.

[6] Wilkinson DJ (2009). Stochastic modelling for quantitative description of heterogeneous biological systems. Nat. Rev. Genet..

[7] Bomze Y, Hey R, Grahn HT, Teitsworth SW (2012). Noise-induced current switching in semiconductor superlattices: Observation of nonexponential kinetics in a high-dimensional system. Phys. Rev. Lett..

[8] Parker M, Kamenev A, Meerson B (2011). Noise-induced stabilization in population dynamics. Phys. Rev. Lett..

[9] Khasin M, Dykman MI (2009). Extinction rate fragility in population dynamics. Phys. Rev. Lett..

[10] Wang G, Zhu X, Gu J, Ao P (2014). Quantitative implementation of the endogenous molecular–cellular network hypothesis in hepatocellular carcinoma. Interface Focus.

[11] Zhu X, Yuan R, Hood L, Ao P (2015). Endogenous molecular-cellular hierarchical modeling of prostate carcinogenesis uncovers robust structure. Prog. Biophys. Mol. Biol..

[12] Wright, S. *The Roles of Mutation*, *Inbreeding*, *Crossbreeding*, *and Selection in Evolution*, vol. 1 (Proceedings of the Sixth International Congress of Genetics, 1932).

[13] Waddington, C. H. *The Strategy of the Genes*, vol. 20 (Allen and Unwin, London, 1957).

[14] Ao P (2004). Potential in stochastic differential equations: novel construction. J. Phys. A.

[15] Wang J, Zhang K, Xu L, Wang E (2011). Quantifying the waddington landscape and biological paths for development and differentiation. Proc. Natl. Acad. Sci. USA.

[16] Elowitz MB, Levine AJ, Siggia ED, Swain PS (2002). Stochastic gene expression in a single cell. Science.

[17] Paulsson J (2004). Summing up the noise in gene networks. Nature.

[18] Choi PJ, Cai L, Frieda K, Xie XS (2008). A stochastic single-molecule event triggers phenotype switching of a bacterial cell. Science.

[19] Lei, X., Tian, W., Zhu, H., Chen, T. & Ao, P. Biological sources of intrinsic and extrinsic noise in cI expression of lysogenic phage lambda. *Sci*. *Rep*. **5** (2015). 10.1038/srep13597 PMC4557085 26329725

[20] Losick R, Desplan C (2008). Stochasticity and cell fate. Science.

[21] Dar RD, Hosmane NN, Arkin MR, Siliciano RF, Weinberger LS (2014). Screening for noise in gene expression identifies drug synergies. Science.

[22] Trendelkamp-Schroer B, Noé F (2016). Efficient estimation of rare-event kinetics. Phys. Rev. X.

[23] Tang Y, Yuan R, Chen J, Ao P (2015). Work relations connecting nonequilibrium steady states without detailed balance. Phys. Rev. E.

[24] Lu M, Onuchic J, Ben-Jacob E (2014). Construction of an effective landscape for multistate genetic switches. Phys. Rev. Lett..

[25] Freidlin, M. I. & Wentzell, A. D. *Random Perturbations of Dynamical Systems*, 3rd edn. (Springer-Verlag, Berlin, 2012).

[26] Zhou JX, Aliyu M, Aurell E, Huang S (2012). Quasi-potential landscape in complex multi-stable systems. J. R. Soc. Interface.

[27] Lv C, Li X, Li F, Li T (2014). Constructing the energy landscape for genetic switching system driven by intrinsic noise. PLoS one.

[28] Wells DK, Kath WL, Motter AE (2015). Control of stochastic and induced switching in biophysical networks. Phys. Rev. X.

[29] Huang S (2009). Non-genetic heterogeneity of cells in development: more than just noise. Development.

[30] Wang G, Zhu X, Hood L, Ao P (2013). From phage lambda to human cancer: endogenous molecular-cellular network hypothesis. Quant. Biol..

[31] Shi J, Chen T, Yuan R, Yuan B, Ao P (2012). Relation of a new interpretation of stochastic differential equations to itô process. J. Stat. Phys..

[32] Yuan R, Ao P (2012). Beyond itô versus stratonovich. J. Stat. Mech..

[33] Strogatz, S. H. *Nonlinear Dynamics and Chaos*: *with Applications to Physics*, *Biology*, *Chemistry*, *and Engineering* (Westview Press, 2014).

[34] Li, S., Zhu, X., Liu, B., Wang, G. & Ao, P. Endogenous molecular network reveals two mechanisms of heterogeneity within gastric cancer. *Oncotarget* (2015). 10.18632/oncotarget.3633 PMC4537037 25962957

[35] Gardiner, C. W. *Handbook of Stochastic Methods*, 3rd edn (Springer-Verlag, Berlin, 2004).

[36] Barkal N, Leibler S (1997). Robustness in simple biochemical networks. Nature.

[37] Gardner TS, Cantor CR, Collins JJ (2000). Construction of a genetic toggle switch in escherichia coli. Nature.

[38] Tyson JJ, Chen KC, Novak B (2003). Sniffers, buzzers, toggles and blinkers: dynamics of regulatory and signaling pathways in the cell. Curr. Opin. Cell Biol..

[39] Wang G (2016). Endogenous network states predict gain or loss of functions for genetic mutations in hepatocellular carcinoma. J. R. Soc. Interface.

[40] Gillespie DT (2000). The chemical langevin equation. J. Chem. Phys..

[41] Das J (2009). Digital signaling and hysteresis characterize ras activation in lymphoid cells. Cell.

[42] Horsthemke, W. & Lefever, R. *Noise*-*Induced Transitions*: *Theory and Applications in Physics*, *Chemistry*, *and Biology*, 2nd edn. (Springer-Verlag, Berlin, 2006).

[43] Sagués F, Sancho JM, Garca-Ojalvo J (2007). Spatiotemporal order out of noise. Rev. Mod. Phys..

[44] Assaf M, Roberts E, Luthey-Schulten Z, Goldenfeld N (2013). Extrinsic noise driven phenotype switching in a self-regulating gene. Phys. Rev. Lett..

[45] Tang Y, Yuan R, Chen J, Ao P (2014). Controlling symmetry-breaking states by a hidden quantity in multiplicative noise. Phys. Rev. E.

[46] Tang Y, Yuan R, Ma Y (2013). Dynamical behaviors determined by the lyapunov function in competitive lotka-volterra systems. Phys. Rev. E.

[47] Yuan R, Wang X, Ma Y, Yuan B, Ao P (2013). Exploring a noisy van der pol type oscillator with a stochastic approach. Phys. Rev. E.

[48] Ma Y, Tan Q, Yuan R, Yuan B, Ao P (2014). Potential function in a continuous dissipative chaotic system: Decomposition scheme and role of strange attractor. Int. J. Bifurcat. Chaos.

[49] Tang Y, Yuan R, Ao P (2014). Summing over trajectories of stochastic dynamics with multiplicative noise. J. Chem. Phys..

[50] Qian H (2014). The zeroth law of thermodynamics and volume-preserving conservative system in equilibrium with stochastic damping. Phys. Lett. A.

[51] Maier RS, Stein DL (1993). Escape problem for irreversible systems. Phys. Rev. E.

[52] Ascher, U. M. & Petzold, L. R. *Computer Methods for Ordinary Differential Equations and Differential*-*Algebraic Equations*, vol. 61 (SIAM, Philadelphia, 1998).

[53] Ge H, Qian H (2012). Landscapes of non-gradient dynamics without detailed balance: Stable limit cycles and multiple attractors. Chaos.

[54] Hanahan D, Weinberg RA (2011). Hallmarks of cancer: the next generation. cell.

[55] Junttila MR, de Sauvage FJ (2013). Influence of tumour micro-environment heterogeneity on therapeutic response. Nature.

[56] Yuan R, Zhu X, Wang G, Li S, Ao P (2017). Cancer as robust intrinsic state shaped by evolution: a key issues review. Rep. Prog. Phys..

[57] MacArthur BD, Ma’ayan A, Lemischka IR (2009). Systems biology of stem cell fate and cellular reprogramming. Nat. Rev. Mol. Cell Biol..

[58] Wust P (2002). Hyperthermia in combined treatment of cancer. Lancet Oncol..

[59] Issels RD (2008). Hyperthermia adds to chemotherapy. Eur. J. Cancer.

[60] Hildebrandt B (2002). The cellular and molecular basis of hyperthermia. Crit. Rev. Oncol. Hematol..

[61] Lançon P, Batrouni G, Lobry L, Ostrowsky N (2001). Drift without flux: Brownian walker with a space-dependent diffusion coefficient. Europhys. Lett..

[62] Volpe G, Helden L, Brettschneider T, Wehr J, Bechinger C (2010). Influence of noise on force measurements. Phys. Rev. Lett..

[63] Raser JM, O’Shea EK (2005). Noise in gene expression: origins, consequences, and control. Science.

[64] Süel GM, Garcia-Ojalvo J, Liberman LM, Elowitz MB (2006). An excitable gene regulatory circuit induces transient cellular differentiation. Nature.

[65] Pedraza JM, Paulsson J (2008). Effects of molecular memory and bursting on fluctuations in gene expression. Science.

[66] Eldar A, Elowitz MB (2010). Functional roles for noise in genetic circuits. Nature.

[67] Gillespie DT (1977). Exact stochastic simulation of coupled chemical reactions. J. Phys. Chem..

[68] Tang Y, Yuan R, Ao P (2015). Anomalous free energy changes induced by topology. Phys. Rev. E.

[69] Tang Y, Yuan R, Ao P (2014). Nonequilibrium work relation beyond the boltzmann-gibbs distribution. Phys. Rev. E.

[70] Vaccario G, Antoine C, Talbot J (2015). First-passage times in *d*-dimensional heterogeneous media. Phys. Rev. Lett..

[71] Zhou P, Li T (2016). Construction of the landscape for multi-stable systems: Potential landscape, quasi-potential, a-type integral and beyond. J. Chem. Phys..

[72] Zhou X, Ren W, E W (2008). Adaptive minimum action method for the study of rare events. J. Chem. Phys..

