# Publisher Correction: Potential landscape of high dimensional nonlinear stochastic dynamics with large noise

**DOI:** 10.1038/s41598-019-47716-1

**Published:** 2019-08-06

**Authors:** Ying Tang, Ruoshi Yuan, Gaowei Wang, Xiaomei Zhu, Ping Ao

**Affiliations:** 1 Department of Physics and Astronomy, Shanghai Jiao Tong University, Shanghai, 200240 China; 2 School of Biomedical Engineering, Shanghai Jiao Tong University, Shanghai, 200240 China; 3 Key Laboratory of Systems Biomedicine Ministry of Education, Shanghai Center for Systems Biomedicine, Shanghai Jiao Tong University, Shanghai, 200240 China; 4 Present Address: Department of Systems Biology, Harvard Medical School, 200 Longwood Avenue, Boston, Massachusetts 02115 USA

Correction to: *Scientific Reports* 10.1038/s41598-017-15889-2, published online 17 November 2017

A supplementary file containing numerical packages was omitted from the original version of the Article. This has been corrected in the HTML version of the Article; the PDF version was correct at time of publication.

