# Supplementary material for: Potential landscape of high dimensional nonlinear stochastic dynamics with large noise
Source: Sci Rep. 2017 Nov 17;7:15762. doi: 10.1038/s41598-017-15889-2 (PMC5693902; doi:10.1038/s41598-017-15889-2)
Supplement: Supplementary file 1 — Supplementary Material [file 41598_2017_15889_MOESM1_ESM.pdf]

Supplementary Material for
"Potential landscape of high dimensional nonlinear stochastic dynamics with large noise"

Ying Tang,[1] Ruoshi Yuan,[2,*] Gaowei Wang,[3] Xiaomei Zhu,[3] and Ping Ao[3,1,†]

[1]*Department of Physics and Astronomy, Shanghai Jiao Tong University, Shanghai 200240, China*
[2]*School of Biomedical Engineering, Shanghai Jiao Tong University, Shanghai 200240, China*
[3]*Key Laboratory of Systems Biomedicine Ministry of Education,*
Shanghai Center for Systems Biomedicine, Shanghai Jiao Tong University, Shanghai 200240, China

(Dated: September 26, 2017)

Contents

I. Analysis on the deviation caused by prevailing stochastic simulations 1
A. Deviation caused by the breaking of the detailed balance condition 1
1. An example with double-well potential 1
B. Deviation caused by multiplicative noise 2
1. An exact example 2

II. "Noise-induced transitions" induced by stochastic integration 3
A. Potential function and steady state distribution 4

III. Action function's forms with different stochastic integrations 4
A. Euler-Lagrangian equation for the least action path 5

IV. Pre-factor of the rate formula due to non-detailed balance part 5

V. Comparison with Freidlin-Wentzell decomposition 6
A. When diffusion matrix is identity 7
B. An example that can be decomposed in our and Freidlin-Wentzell way 7

VI. Another biological example 8
A. Toggle switch 8
B. A model for cell fate decision 8

VII. ODEs for the 38 dimensional prostate cancer model 10

References 12

I. ANALYSIS ON THE DEVIATION CAUSED BY PREVAILING STOCHASTIC SIMULATIONS

A. Deviation caused by the breaking of the detailed balance condition

1. An example with double-well potential

We demonstrate that the deviation between ODE and SDE can be caused by breakdown of detailed balance ($Q(\mathbf{x}) \neq 0$), and it happens even for examples with additive noise. Under the circumstances, conventional stochastic integrations like Ito's and Stratonovich's show no difference, but they are distinct from A-type due to the non-detailed balance part $Q(\mathbf{x})$.

---

*The first two authors contributed equally to this paper.
†Electronic address: aoping@sjtu.edu.cn

TABLE I: Potential difference between the saddle point and the two stable fixed points for the example Eq. (1) with multiplicative noise. The values are obtained by least action method, where we set $K = 500$, and do minimization for $T \in [0.5,\ 10]$ with 0.5 as step length. We choose the saddle point as the zero potential reference. The other parameters are $d = a = H = 1$.

Fixed points	(0,1)	(1,1)	(2,1)
$d_1 = 0, d_2 = 0$	-0.2495	0	-0.2418
$d_1 = 1, d_2 = 10$	-0.2502	0	-0.2485
$d_1 = 10, d_2 = 10$	-0.2514	0	-0.2432

The example is given by:

$$\begin{cases} \dot{x}_1 = -dH[-(x_1-1)+(x_1-1)^3] \\ \qquad - ax_1(x_2-3)H(x_2-1) + \sqrt{d} * \zeta_{x_1}(t), \\ \dot{x}_2 = ax_1(x_2-3)H[-(x_1-1)+(x_1-1)^3] \\ \qquad - dH(x_2-1) + \sqrt{d} * \zeta_{x_2}(t). \end{cases}$$

It can be rewritten in the form of Eq. (4) of main text with the potential function $\phi(x_1, x_2) = H[(x_2-1)^2/2 - (x_1-1)^2/2 + (x_1-1)^4/4]$ having double-well in the x_1 direction, and:

$$D = dI, \quad Q = ax_1(x_2-3)\begin{pmatrix} 0 & 1 \\ -1 & 0 \end{pmatrix}. \tag{1}$$

The parameters are d, a, H. The system has a saddle point $\mathbf{s}^* = (1,1)$, and two stable fixed points $\mathbf{x}_1^* = (2,1), \mathbf{x}_2^* = (0,1)$. Their potential values by the analytical formula are: $\phi|_{\mathbf{s}^*} = 0$, $\phi|_{\mathbf{x}_1^*} = \phi|_{\mathbf{x}_2^*} = -H/4$.

We simulate SDE with both A-type and Ito's interpretation. Simulation under Ito's integration leads to deviation on the position of the potential minimum and the potential height [1], as shown in FIG. 1 of main text (a-c). To realize A-type interpretation, we need to add the term $\epsilon\partial_{x_j}Q_{ij}(\mathbf{x}) = \epsilon a(x_1, -(x_2-3))^\tau$ to the drift and use the equivalent Ito's interpretation:

$$\begin{cases} \dot{x}_1 = -dH[-(x_1-1)+(x_1-1)^3] \\ \qquad - ax_1(x_2-3)H(x_2-1) + \epsilon a x_1 + \sqrt{d} \cdot \zeta_{x_1}(t), \\ \dot{x}_2 = ax_1(x_2-3)H[-(x_1-1)+(x_1-1)^3] \\ \qquad - dH(x_2-1) - \epsilon a(x_2-3) + \sqrt{d} \cdot \zeta_{x_2}(t). \end{cases}$$

Then, the potential function is obtained by simulating the steady state distribution with A-type integration $\phi = \epsilon \ln \rho|_A$, and it is quantitatively consistent with the analytical formula, as shown in FIG. 1 of main text (d-e). Note that the matrix $Q(\mathbf{x})$ leads to different simulation results between Ito's and A-type.

We also use the least action method to calculate the potential values of points along the line $x_1 = 1$, as shown in FIG. 1 of main text (f). The potential differences by the least action method and A-type simulation are quantitatively consistent with the analytical formula, whereas Ito's simulation causes deviation on positions of fixed points and barrier height.

We further consider this system with multiplicative noise. For clarity, we choose:

$$D = \begin{pmatrix} d + d_1 x_1^2 & 0 \\ 0 & d + d_2 x_2^2 \end{pmatrix}, \tag{2}$$

where the two parameters d_1 and d_2 are nonzero. We obtain potential differences between the saddle point and the two stable fixed points for different d_1, d_2. The potential values as listed in table I are also quantitatively consistent with the analytical formula. Systems with more general diffusion matrix $D(\mathbf{x})$ can be studied similarly.

B. Deviation caused by multiplicative noise

1. *An exact example*

It is widely known that in the presence of multiplicative noise different stochastic integrations of Eq. (2) of main text lead to distinct results. Typically, Ito's or Strantonovich's integration was used [2]. However, recent works have

demonstrated applications of stochastic integrations beyond Ito-Stratonovich both theoretically [1] and experimentally [3, 4]. Here, we show analytically that Ito's integration causes deviation compared with dynamics of deterministic counterpart, but A-type gives consistent results.

We consider a set of SDE with multiplicative noise:

$$\begin{cases} \dot{x}_1 &= 2x_1 - x_1\left(x_1^2+x_2^2\right) + \sqrt{x_1^2+x_2^2}\zeta_{x_1}(t) \\ \dot{x}_2 &= 2x_2 - x_2\left(x_1^2+x_2^2\right) + \sqrt{x_1^2+x_2^2}\zeta_{x_2}(t) \end{cases} \tag{3}$$

where $\epsilon = 1$, the diffusion matrix $D(x_1, x_2) = \left(x_1^2 + x_2^2\right) I$, and the stochastic integration has not been specified. Under A-type integration, the steady state distribution of this system is $\rho_{ss}(\mathbf{x})|_A = \exp[-\phi(\mathbf{x})/\epsilon]/Z_A$ with the normalization constant Z_A and

$$\phi(\mathbf{x}) = -\ln\left(x_1^2+x_2^2\right) + \frac{x_1^2+x_2^2}{2} \ . \tag{4}$$

The distribution of Ito's simulation for Eq. (3) is identical to the A-type's simulation for the system:

$$\begin{cases} \dot{x}_1 &= -x_1\left(x_1^2+x_2^2\right) + \sqrt{x_1^2+x_2^2} * \zeta_{x_1}(t) \\ \dot{x}_2 &= -x_2\left(x_1^2+x_2^2\right) + \sqrt{x_1^2+x_2^2} * \zeta_{x_2}(t) \end{cases} \tag{5}$$

whose expression can be similarly calculated as $\rho_{ss}(\mathbf{x})|_I = \exp[-\psi(\mathbf{x})/\epsilon]/Z_I$ with with the normalization constant Z_I and

$$\psi(\mathbf{x}) = \frac{1}{2}\left(x_1^2+x_2^2\right) \ . \tag{6}$$

The two distributions $\rho_{ss}(\mathbf{x})|_A$ and $\rho_{ss}(\mathbf{x})|_I$ have obvious differences, for instance, $\rho_{ss}(0,0)|_A = 0$ but the origin $(0,0)$ is the most probable state for $\rho_{ss}(\mathbf{x})|_I$.

II. "NOISE-INDUCED TRANSITIONS" INDUCED BY STOCHASTIC INTEGRATION

We demonstrate here that occurrence of the noise-induced transitions [5–8] depends on which deterministic dynamics to compare with. The deterministic ODE counterpart obtained from the Fokker-Planck equation is dependent on choosing the stochastic integration, and the present A-type integration leads to a consistent result between SDE and its ODE counterpart. This raises the question on how to choose a proper mean field deterministic dynamics for a given chemical reaction scheme: 1) directly using the rate equations or, 2) starting from master equation, expanding as Fokker-Planck equation, getting a SDE with A-type integration, and taking the corresponding ODE.

We consider the system in [8] as an example. We start from the Fokker-Planck equation obtained there:

$$\partial_t\rho(x_1,x_2,t) = \Big[-\partial_{x_1}\mathcal{A}_1 - \partial_{x_2}\mathcal{A}_2 + \frac{1}{2N}\sum_{i,j=1}^{2}\partial_{x_i}\partial_{x_j}\mathcal{B}_{ij}\Big]\rho(x_1,x_2,t), \tag{7}$$

where $\mathcal{A}_1 = -\mathcal{A}_2 = \varepsilon(x_2 - x_1)$ and $\mathcal{B}_{ij} = (2rx_1x_2 + \varepsilon(x_1+x_2))(-1)^{i+j}$. After doing the coordinate transformation:

$$\begin{cases} w &= x_1 + x_2, \\ z &= x_1 - x_2, \end{cases} \quad \begin{cases} x_1 &= (w+z)/2, \\ x_2 &= (w-z)/2, \end{cases} \tag{8}$$

with the Jacobian $J = 1/2$ and $\tilde{J} = 2$, the transformed Fokker-Planck equation is one-dimensional:

$$\partial_t\tilde{\rho}(w,z,t) = \Big\{-\partial_z(-2\varepsilon z) + \frac{1}{2N}\partial_z^2[r(w^2-z^2)+2\varepsilon w]\Big\}\tilde{\rho}(w,z,t). \tag{9}$$

Thus, the diffusion coefficient is:

$$D(w,z) = \frac{1}{2N}[r(w^2-z^2)+2\varepsilon w], \tag{10}$$

with $\partial_z D(w,z) = -rz/N$.

For a given FPE, one can derive a class of corresponding SDEs with using various stochastic integrations. If A-type integration is used, we get a SDE whose ODE part is consistent with the underlying FPE dynamics. In detail, with A-type integration rule [1], we obtain the stochastic differential equation:

$$\dot{z} = -2\varepsilon z + \frac{1}{N} r z + \sqrt{D(w,z)} * \zeta(t), \tag{11}$$

where the asterisk denotes the A-type integration, $\zeta(t)$ is Gaussian white noise with $\langle \zeta(t) \rangle = 0$, $\langle \zeta(t)\zeta^\tau(t^{'}) \rangle = 2\epsilon\delta(t-t^{'})$. Here the superscript τ denotes transpose, $\delta(t-t^{'})$ is the Dirac delta function, and $\langle \cdots \rangle$ represents noise average.

According to [8], we set $r=1$ without loss of generality, because we can rescale ε to absorb r. As the total number of ants N is conserved, we have $w=1$. Thus, $D(w,z) = [(1-z^2)+2\varepsilon]/2N$, and Eq. (11) becomes:

$$\dot{z} = \left(\frac{1}{N} - 2\varepsilon\right) z + \sqrt{\frac{1}{2N}[(1-z^2)+2\varepsilon]} * \zeta(t), \tag{12}$$

With using the Ito's integration rule [2], the corresponding stochastic differential equation is:

$$\dot{z} = -2\varepsilon z + \sqrt{\frac{1}{2N}[(1-z^2)+2\varepsilon]} \cdot \zeta(t), \tag{13}$$

where the dot denotes the Ito's integration.

Note that the drift terms for the above two SDEs are different. The variable $z = x_1 - x_2$ ranges over the interval $[-1,1]$. Then, the deterministic force in Eq. (12) can both push the system away from the state $z^* = 0$ when $1 > 2\varepsilon N$, and attract the system back to $z^* = 0$ when $1 < 2\varepsilon N$. Therefore, ODE part in Eq. (12) is consistent with the underlying FPE dynamics.

A. Potential function and steady state distribution

The potential function satisfying Eq. (12) is:

$$\phi(z) = (1 - 2N\varepsilon)\ln(1 + 2\varepsilon - z^2), \tag{14}$$

and steady state obeys Boltzmann-Gibbs distribution:

$$\rho(z) \doteq \frac{1}{Z}\exp[-\phi(z)] = \frac{1}{Z(1+2\varepsilon-z^2)^{(1-2N\varepsilon)}}, \tag{15}$$

where Z is normalization constant. When $1 > 2N\varepsilon$, the distribution has a U shape, and the system is bistable. When $1 < 2N\varepsilon$, the distribution has an inverted U shape, and the distribution is centered at $z^* = 0$. Thus, the deterministic force in Eq. (12) shows consistent behaviors as the steady state distribution.

III. ACTION FUNCTION'S FORMS WITH DIFFERENT STOCHASTIC INTEGRATIONS

The difference of action functions and least action paths under various stochastic integrations, e.g. A-type, Ito's and Stratonovich's, can be neglected in the limit of $\epsilon \to 0$, which can be proved similarly as the above procedure in Eq. (9) of main text. Take the action function Stratonovich's integration as an example,

$$\begin{aligned} S_T[\mathbf{x}]|_S &= \frac{1}{4}\int_{T_1}^{T_2} \Big|_S dt(\dot{\mathbf{x}} - \mathbf{f})^\tau D^{-1}(\dot{\mathbf{x}} - \mathbf{f}) + \frac{1}{2}\epsilon J_S \\ &= \frac{1}{4}\int_{T_1}^{T_2} \Big|_I dt[\dot{\mathbf{x}} - D\nabla\phi + Q\nabla\phi]^\tau D^{-1} \\ &\quad \times [\dot{\mathbf{x}} - D\nabla\phi + Q\nabla\phi] + \int_{T_1}^{T_2} \Big|_S dt\dot{\mathbf{x}}^\tau \nabla\phi + \frac{1}{2}\epsilon J_S \\ &\geq \Delta\phi + \frac{1}{2}\epsilon J_S, \end{aligned} \tag{16}$$

TABLE II: action functions for different stochastic integrations (A-type, Ito's and Stratonovich's) and their transformed forms with Ito's interpretation. The usual Freidlin-Wentzell's action [10] is the same as the Ito's form here. The order for the time integral of the Jacobian term J_A is $\mathcal{O}(1)$. The difference of action functions among these stochastic integrations can be neglected in the limit of $\epsilon \to 0$.

Stochastic integration	The present action function $S_T[\mathbf{x}]$	$S_T[\mathbf{x}]$ transformed to Ito's interpretation
A-type	$\frac{1}{4}\int_{T_1}^{T_2}\vert_A dt(\dot{\mathbf{x}}-\mathbf{f})^\tau D^{-1}(\dot{\mathbf{x}}-\mathbf{f})+\epsilon J_A$	$\frac{1}{4}\int_{T_1}^{T_2}\vert_I dt[\dot{\mathbf{x}}-\mathbf{f}-\epsilon\Delta\mathbf{f}]^\tau D^{-1}[\dot{\mathbf{x}}-\mathbf{f}-\epsilon\Delta\mathbf{f}]$
Ito's (Freidlin-Wentzell)	$\frac{1}{4}\int_{T_1}^{T_2}\vert_I dt(\dot{\mathbf{x}}-\mathbf{f})^\tau D^{-1}(\dot{\mathbf{x}}-\mathbf{f})$	$\frac{1}{4}\int_{T_1}^{T_2}\vert_I dt(\dot{\mathbf{x}}-\mathbf{f})^\tau D^{-1}(\dot{\mathbf{x}}-\mathbf{f})$
Stratonovich's	$\frac{1}{4}\int_{T_1}^{T_2}\vert_S dt(\dot{\mathbf{x}}-\mathbf{f})^\tau D^{-1}(\dot{\mathbf{x}}-\mathbf{f})+\frac{1}{2}\epsilon J_S$	$\frac{1}{4}\int_{T_1}^{T_2}\vert_I dt(\dot{\mathbf{x}}-\mathbf{f}-\epsilon\nabla D/2)^\tau D^{-1}(\dot{\mathbf{x}}-\mathbf{f}-\epsilon\nabla D/2)$

where J_S is the time integral of the Jacobian term. In the limit of $\epsilon \to 0$, $S_T[\mathbf{x}]|_S \geq \Delta\phi(\mathbf{x})|_{\mathbf{x}^*}^{\mathbf{s}^*}$. The typical forms of action function and their transformed form with Ito's interpretation [9] are listed in table II. The discretized scheme when doing numerical minimization should corresponds to their stochastic integration, for example, pre-point scheme is needed for Ito's interpretation. Because the difference among stochastic integrations is negligible in the limit of $\epsilon \to 0$, we can choose convenient schemes as needed.

A. Euler-Lagrangian equation for the least action path

Euler-Lagrangian equation from the action Eq. (9) in main text is calculate as follows. For convenience, we rewrite the Lagrangian to be:

$$L = \frac{1}{4}(\dot{x}_i - f_i)D_{ij}^{-1}(\dot{x}_j - f_j), \tag{17}$$

where we have used the notion of Einstein summation in this paper. Then, the Euler-Lagrangian equation is an ordinary differential equation:

$$\begin{aligned} 0 &= \frac{d}{dt}\frac{\partial L}{\partial \dot{x}_l} - \frac{\partial L}{\partial x_l} \\ &= \frac{1}{2}\Big[\frac{\partial D_{lj}^{-1}}{\partial x_i}\dot{x}_i(x_j - f_j) + D_{lj}^{-1}\Big(\ddot{x}_j - \frac{\partial f_j}{\partial x_i}\dot{x}_i\Big)\Big] \\ &\quad + \frac{1}{2}\frac{\partial f_i}{\partial x_l}D_{ij}^{-1}(\dot{x}_j - f_j) - \frac{1}{4}(\dot{x}_i - f_i)\frac{\partial D_{ij}^{-1}}{\partial x_l}(\dot{x}_j - f_j), \end{aligned} \tag{18}$$

which gives the solution of the least action path.

IV. PRE-FACTOR OF THE RATE FORMULA DUE TO NON-DETAILED BALANCE PART

The FPE corresponding to Eq. (4) of main text is [1]:

$$\partial_t \rho(\mathbf{x}, t) = \nabla_{\mathbf{x}}^\tau [D(\mathbf{x}) + Q(\mathbf{x})][\nabla_{\mathbf{x}}\phi(\mathbf{x}) + \nabla_{\mathbf{x}}]\rho(\mathbf{x}, t), \tag{19}$$

where the steady state obeys Boltzmann-Gibbs distribution $\rho(\mathbf{x}, t \to \infty) = \exp[-\phi(\mathbf{x})/\epsilon]$.

Inserting WKB ansatz with pre-factor:

$$\rho_{ss}(\mathbf{x}) = C(\mathbf{x})\exp\Big(-\frac{\phi(\mathbf{x})}{\epsilon}\Big) \tag{20}$$

into our FPE at steady state:

$$\begin{aligned} 0 &= \epsilon\nabla_{\mathbf{x}}^\tau [D(\mathbf{x}) + Q(\mathbf{x})][\nabla_{\mathbf{x}} C(\mathbf{x})]\exp\Big(-\frac{\phi(\mathbf{x})}{\epsilon}\Big) \\ &\propto \epsilon\{\nabla_{\mathbf{x}}^\tau [D(\mathbf{x}) + Q(\mathbf{x})]\}[\nabla_{\mathbf{x}} C(\mathbf{x})] + \epsilon[D(\mathbf{x}) + Q(\mathbf{x})]\cdot[\nabla_{\mathbf{x}}^\tau \nabla_{\mathbf{x}} C(\mathbf{x})] - [\nabla_{\mathbf{x}}^\tau \phi(\mathbf{x})][D(\mathbf{x}) + Q(\mathbf{x})][\nabla_{\mathbf{x}} C(\mathbf{x})], \end{aligned} \tag{21}$$

we get for the order of $\mathcal{O}(1)$:

$$0 = [\nabla_{\mathbf{x}}^{\tau}\phi(\mathbf{x})][D(\mathbf{x}) + Q(\mathbf{x})][\nabla_{\mathbf{x}}C(\mathbf{x})], \tag{22}$$

which implies that $C(\mathbf{x})$ is a constant. Note that we does not have the order of $\mathcal{O}(1/\epsilon)$ here, for which the Hamilton-Jacobi equation

$$\nabla_{\mathbf{x}}^{\tau}\phi(\mathbf{x})D(\mathbf{x})\nabla_{\mathbf{x}}\phi(\mathbf{x}) + \nabla_{\mathbf{x}}^{\tau}\phi(\mathbf{x})\mathbf{f}(\mathbf{x}) = 0 \tag{23}$$

is obtained by the decomposition [11].

We also insert the WKB ansatz to the FPE corresponding to Eq. (6) of main text:

$$\begin{aligned}
0 = &-\{\nabla_{\mathbf{x}}^{\tau}[\mathbf{f}(\mathbf{x}) + \epsilon\Delta\mathbf{f}(\mathbf{x})]\}C(\mathbf{x}) + \epsilon[\nabla_{\mathbf{x}}^{\tau}\nabla_{\mathbf{x}}D(\mathbf{x})]C(\mathbf{x}) - \Big[\nabla_{\mathbf{x}}^{\tau}C(\mathbf{x}) - \frac{1}{\epsilon}C(\mathbf{x})\nabla_{\mathbf{x}}^{\tau}\phi(\mathbf{x})\Big][\mathbf{f}(\mathbf{x}) + \epsilon\Delta\mathbf{f}(\mathbf{x})] \\
&+ 2\epsilon\nabla_{\mathbf{x}}^{\tau}D(\mathbf{x})\Big[\nabla_{\mathbf{x}}C(\mathbf{x}) - \frac{1}{\epsilon}C(\mathbf{x})\nabla_{\mathbf{x}}\phi(\mathbf{x})\Big] \\
&+ \epsilon D(\mathbf{x})\Big[\nabla_{\mathbf{x}}^{\tau}\nabla_{\mathbf{x}}C(\mathbf{x}) - \frac{2}{\epsilon}\nabla_{\mathbf{x}}^{\tau}\phi(\mathbf{x})\nabla_{\mathbf{x}}C(\mathbf{x}) - \frac{1}{\epsilon}C(\mathbf{x})\nabla_{\mathbf{x}}^{\tau}\nabla_{\mathbf{x}}\phi(\mathbf{x}) - \frac{1}{\epsilon^2}C(\mathbf{x})\nabla_{\mathbf{x}}^{\tau}\phi(\mathbf{x})\nabla_{\mathbf{x}}\phi(\mathbf{x})\Big],
\end{aligned} \tag{24}$$

where $\Delta f_i(\mathbf{x}) = \partial_{x_j}[D_{ij}(\mathbf{x}) + Q_{ij}(\mathbf{x})]$. Then, we get Eq. (23) for the order of $\mathcal{O}(1/\epsilon)$, and for the order of $\mathcal{O}(1)$:

$$0 = -\nabla_{\mathbf{x}}^{\tau}\phi(\mathbf{x})[D(\mathbf{x}) + Q(\mathbf{x})]\nabla_{\mathbf{x}}C(\mathbf{x}), \tag{25}$$

which again implies that $C(\mathbf{x})$ is a constant. The order of $\mathcal{O}(\epsilon)$ is also zero. As a result, the non-detailed balance part does not provide correction terms that explicitly appear in the pre-factor of the rate formula.

In general, the pre-factor in the rate formula depends on other coefficients such as the friction coefficient [12, 13], and from Kramers equation its dependence on the friction coefficient is significantly different for large friction and small friction limit.

V. COMPARISON WITH FREIDLIN-WENTZELL DECOMPOSITION

Our decomposition on the drift force $\mathbf{f}(\mathbf{x}) = -[D(\mathbf{x}) + Q(\mathbf{x})]\nabla\phi(\mathbf{x})$ with $\nabla\phi(\mathbf{x})Q\nabla\phi(\mathbf{x}) = 0$ is generally different form the usual Freidlin-Wentzell form $\mathbf{f}(\mathbf{x}) = -\nabla U(\mathbf{x}) + l(\mathbf{x})$ with $\nabla U(\mathbf{x}) \cdot l(\mathbf{x}) = 0$ [10], based on which landscape has been calculated in various systems recently [14–16]. They are mathematically identical when the diffusion matrix $D(\mathbf{x})$ is identity. For general diffusion matrix $D(\mathbf{x})$, the action function Eq. (11) in the main text does not directly equal to the potential function $U(\mathbf{x})$ in Freidlin-Wentzell decomposition. We demonstrate it more clearly by a simplified case as follows.

We show that when diffusion matrix is a diagonal matrix with distinct constant elements, the Freidlin-Wentzell action does not give the exact potential difference even with $\epsilon \to 0$. If we have $\mathbf{f}(\mathbf{x}) = -\nabla U(\mathbf{x}) + l(\mathbf{x})$ with $\nabla U(\mathbf{x}) \cdot l(\mathbf{x}) = 0$, then

$$\begin{aligned}
S_T[\mathbf{x}]|_I &= \sum_i \int_{T_1}^{T_2} \Big|_I dt \frac{1}{4D_{ii}}(\dot{x}_i - f_i)^2 \\
&= \sum_i \int_{T_1}^{T_2} \Big|_I dt \frac{1}{4D_{ii}}(\dot{x}_i + \partial_{x_i}U - l_i)^2 \\
&\geq \sum_i \int_{T_1}^{T_2} \Big|_I dt \frac{1}{D_{ii}}\dot{x}_i\partial_{x_i}U - \sum_i \int_{T_1}^{T_2} \Big|_I dt \frac{\partial_{x_i}U}{D_{ii}} l_i,
\end{aligned} \tag{26}$$

where the last term cannot be eliminated when $\epsilon \to 0$.

On the other hand, if we apply the action without the element of diffusion matrix [15],

$$S_T[\mathbf{x}] = \sum_i \int_{T_1}^{T_2} dt \frac{1}{4}(\dot{x}_i - f_i)^2, \tag{27}$$

it does not count the effect of diffusion matrix explicitly.

A. When diffusion matrix is identity

When diffusion matrix is identity, our decomposition is the same as the usual Freidlin-Wentzell form, and both action functions give the potential function. Besides, various stochastic interpretations give the same result when ϵ is small. The drift force in the stochastic differential equation (SDE) of the main text has a decomposition $\mathbf{f}(\mathbf{x}) = -\nabla U(\mathbf{x}) + l(\mathbf{x})$ with $\nabla U(\mathbf{x}) \cdot l(\mathbf{x}) = 0$. For convenience, we use the equivalent action function under Ito's interpretation:

$$
\begin{aligned}
S_T[\mathbf{x}]|_I &= \sum_i \int_{T_1}^{T_2} \Big|_I dt \frac{1}{4}(\dot{x}_i - f_i)^2 \\
&= \sum_i \int_{T_1}^{T_2} \Big|_I dt \frac{1}{4}(\dot{x}_i + \partial_{x_i} U - l_i)^2 \\
&\geq \Delta U - \epsilon \sum_i \int_{T_1}^{T_2} \Big|_I dt \partial_{x_i}^2 U,
\end{aligned} \tag{28}
$$

where the subscript I means Ito's interpretation. If we can get a special trajectory satisfying $\dot{\mathbf{x}} = \nabla U(\mathbf{x}) + l(\mathbf{x})$, the equality holds. The equality relies on the crucial condition $\nabla U(\mathbf{x}) \cdot l(\mathbf{x}) = 0$. The minimized action gives ΔU when $\epsilon \to 0$

B. An example that can be decomposed in our and Freidlin-Wentzell way

We construct an example that can be decomposed as both our ways and Freidlin-Wentzell way. It is given by a SDE under A-type integration:

$$
\begin{cases}
\dot{x}_1 = -(1 + x_1^2)x_1 - [1 - x_1 x_2 - (x_2^2 - x_1^2)]x_2 + \zeta_{x_1}(t), \\
\dot{x}_2 = -[1 - x_1 x_2 + (x_2^2 - x_1^2)]x_1 - (1 + x_2^2)x_2 + \zeta_{x_2}(t),
\end{cases} \tag{29}
$$

where $\langle \zeta(t) \rangle = 0$, $\langle \zeta(t) \zeta^\tau(t') \rangle = 2\epsilon\delta(t - t')$. We have:

$$
\begin{aligned}
D &= \begin{pmatrix} 1 + x_1^2 & 1 - x_1 x_2 \\ 1 - x_1 x_2 & 1 + x_2^2 \end{pmatrix} \geq 0, \\
Q &= \begin{pmatrix} 0 & -(x_2^2 - x_1^2) \\ (x_2^2 - x_1^2) & 0 \end{pmatrix}, \quad \phi = (x_1^2 + x_2^2)/2.
\end{aligned} \tag{30}
$$

Note that $D(\mathbf{x})$ and $Q(\mathbf{x})$ are singular along the line $x_2 = -x_1$. This singularity leads to nonzero finite potential values at the fixed points along $x_2 = -x_1$ for Eq. (29) without noise.

The system can also be decomposed as:

$$
\begin{cases}
\dot{x}_1 = -(x_1 + x_2) - (x_1^3 + x_1^2 x_2 - x_1 x_2^2 - x_2^3) + \zeta_{x_1}(t), \\
\dot{x}_2 = -(x_1 + x_2) - (-x_1^3 - x_1^2 x_2 + x_1 x_2^2 + x_2^3) + \zeta_{x_2}(t).
\end{cases} \tag{31}
$$

Thus, we get $\mathbf{f}(\mathbf{x}) = -\nabla U(\mathbf{x}) + l(\mathbf{x})$ with

$$
U = (x_1^2 + x_2^2)/2 + x_1 x_2, \quad l = \begin{pmatrix} -(x_1 + x_2)(x^2 - x_2^2) \\ (x_1 + x_2)(x^2 - x_2^2) \end{pmatrix}, \tag{32}
$$

and $\nabla^\tau U(\mathbf{x}) \cdot l(\mathbf{x}) = 0$. Note that minimum of $U(\mathbf{x})$ corresponds to the fixed points of Eq. (29) without noise.

From the above derivation, we see that for the corresponding deterministic system the potential function constructed in our framework is not unique without given a diffusion matrix $D(\mathbf{x})$.

TABLE III: Potential barrier between the three stable fixed points calculated by the least action method for the example Eq. (34). The mesendodermal state, the ectodermal state, and the pluripotency state, representing high concentration of the corresponding genes, are given by the three stable fixed points $\mathbf{x}_1^* = (0.0218, 0.0218, 1, 0.0545)$, $\mathbf{x}_2^* = (0.0218, 0.0218, 0.0545, 1)$, $\mathbf{x}_3^* = (1.3309, 1.3309, 0.4423, 0.4423)$. We set $K = 100$, $T = 50$. We have checked that larger K and T values lead to convergent results with relative error smaller than 0.005. The parameters of the system are the same as those in [18].

Initial states	mesendodermal		ectodermal		pluripotency	
End states	ectodermal	pluripotency	mesendodermal	pluripotency	mesendodermal	ectodermal
Potential barrier	0.0736	0.0697	0.0736	0.0699	0.0318	0.0332

VI. ANOTHER BIOLOGICAL EXAMPLE

A. Toggle switch

We first consider the dynamical system describing the genetic toggle switch [17] with additive noise. The deterministic part of the dynamical model is:

$$\begin{cases} \dot{x}_1 = \dfrac{\alpha_1}{1 + x_2^{\beta}} - x_1, \\ \dot{x}_2 = \dfrac{\alpha_2}{1 + x_2^{\gamma}} - x_2. \end{cases} \tag{33}$$

where x_1 and x_2 are the concentration of the Repressor 1 and 2 separately, and α_1, α_2, β, γ are parameters with specific biological meaning. The noise is Gaussian and white. Here, we consider the parameters $\alpha_1 = \alpha_2 = \beta = 2$, $\gamma = 6$, and the diffusion matrix is set as $D(x_1, x_2) = I$ with identity matrix I. By analyzing the structure of nullclines ($dx/dt = 0$ and $dy/dt = 0$), bistability happens when β, $\gamma > 1$ [17]. The two states Repressor 1 and Repressor 2, denoting high concentration of the corresponding repressor, are given by stable fixed points calculated from ODE $\mathbf{x}_1^* = (1.9981, 0.0309)$, $\mathbf{x}_2^* = (0.4027, 1.9915)$.

We use Ito's simulation to get the steady state distribution. In FIG. 1, Ito's simulation leads to deviation on the position of the potential minimum. When noise strength is large, Ito's simulation show that one stable state is destroyed in the bistable parameter region of ODE. Note that Stratonovich's and Ito's integrations generate identical results for Eq. (33) with additive noise.

The potential landscape is obtained by the Protocol II with the least action method, as shown in FIG. 2. The potential difference from Repressor 1 to Repressor 2 is 0.2819, which is greater than that from the other way around 0.1353. It demonstrates that the genetic switch prefers the state with higher concentration of Repressor 1 under the given parameters. Therefore, the result on potential barrier height tells the relative stability. Besides, the steady state given by the Boltzmann-Gibbs distribution $\rho_{ss}(\mathbf{x}) \propto \exp[-\phi(\mathbf{x})/\epsilon]$ under various noise strengths show that the bistable dynamical structure is preserved by using A-type integration. The cases with other parameters can be calculated similarly

B. A model for cell fate decision

We apply our method to a 4 dimensional model which was used to study cell fate determination in pluripotent stem or progenitor cells [18]. This model consists of two coupled modules: the pluripotency module and the differentiation module. The pluripotency module is represented by the mutual activation of Oct4 and Sox2, whereas the differentiation module is modeled by mutually inhibiting mesendodermal and ectodermal genes. Inhibitions or activations among these nodes were given in [18]. A quantitative description of the model consists of a set of coupled stochastic differential

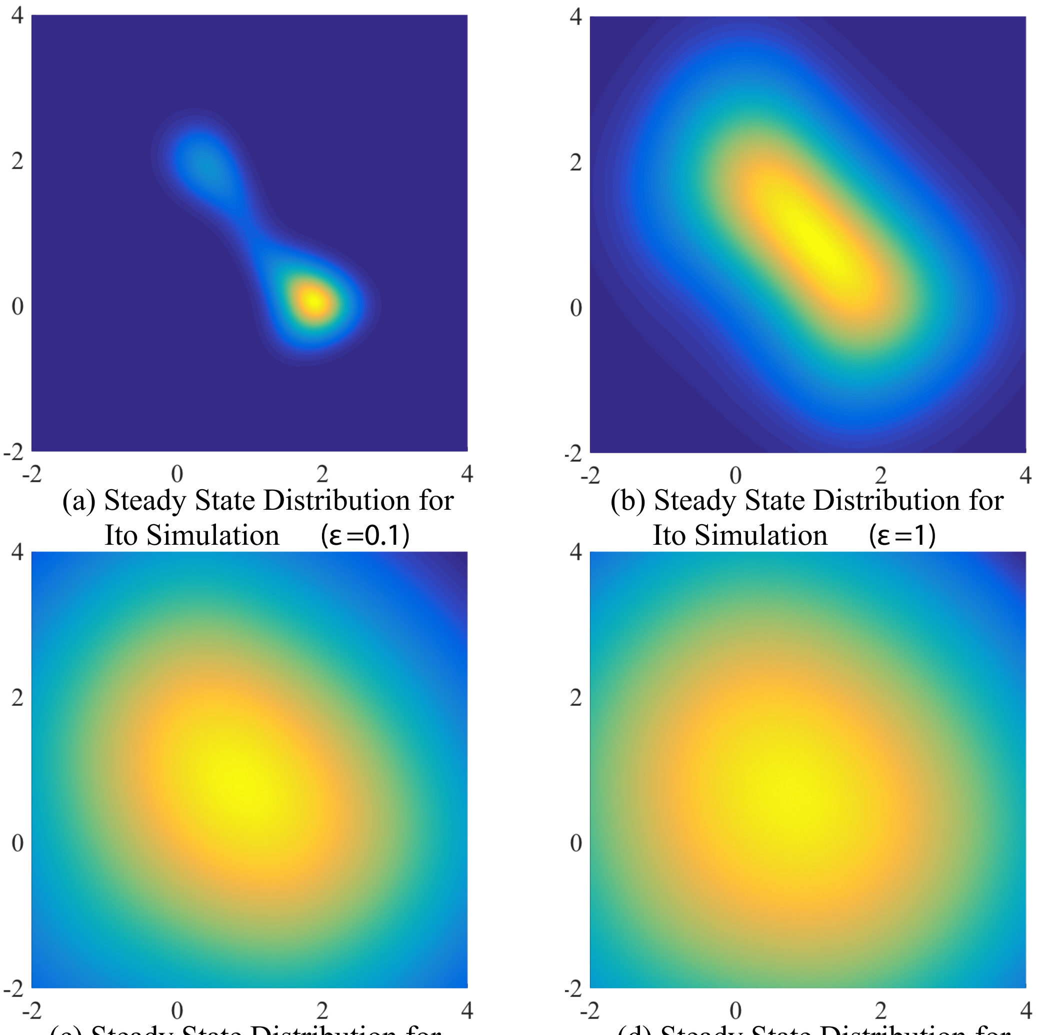

(a) Steady State Distribution for Ito Simulation (ε=0.1)

(b) Steady State Distribution for Ito Simulation (ε=1)

(c) Steady State Distribution for Ito Simulation (ε=5)

(d) Steady State Distribution for Ito Simulation (ε=10)

FIG. 1: (Color online) Stability structure of steady state distribution is altered when noise increases. The example is given by Eq. (33) with additive noise, where Ito's simulation leads to deviation on the positions of the locally most probable states. When noise strength is large, one of the stable states is destroyed in the bistable parameter region of ODE. The noise strengths are $\epsilon = 0.1, 1, 5, 10$.

equations with additive noise. The deterministic part is given by ODEs:

$$\begin{cases} \dot{x}_1 = d_0\Big[C_0 + I_0\dfrac{K_{p\_inh}^{n_{p\_inh}}}{K_{p\_inh}^{n_{p\_inh}} + x_3^{n_{p\_inh}}}\dfrac{K_{p\_inh}^{n_{p\_inh}}}{K_{p\_inh}^{n_{p\_inh}} + x_4^{n_{p\_inh}}} \cdot \Big(KM + \dfrac{(Oct4 \cdot Sox2)^{n_{p\_act}}}{K_{p\_act}^{n_{p\_act}} + (Oct4 \cdot Sox2)^{n_{p\_act}}}\Big) - x_1\Big], \\ \dot{x}_2 = d_s\Big[C_s + I_s\dfrac{K_{p\_inh}^{n_{p\_inh}}}{K_{p\_inh}^{n_{p\_inh}} + x_3^{n_{p\_inh}}}\dfrac{K_{p\_inh}^{n_{p\_inh}}}{K_{p\_inh}^{n_{p\_inh}} + x_4^{n_{p\_inh}}} \cdot \Big(KM + \dfrac{(Oct4 \cdot Sox2)^{n_{p\_act}}}{K_{p\_act}^{n_{p\_act}} + (Oct4 \cdot Sox2)^{n_{p\_act}}}\Big) - x_2\Big], \\ \dot{x}_3 = d_M\Big[C_M + I_M w\dfrac{x_1^{n_a}}{K_a^{n_a} + x_1^{n_a}} + I_M\dfrac{K_i^{n_i}}{K_i^{n_i} + x_2^{n_i}}\dfrac{K_{d\_inh}^{n_{d\_inh}}}{K_{d\_inh}^{n_{d\_inh}} + x_4^{n_{d\_inh}}} - x_3\Big], \\ \dot{x}_4 = d_E\Big[C_E + I_E w\dfrac{x_2^{n_a}}{K_a^{n_a} + x_2^{n_a}} + I_M\dfrac{K_i^{n_i}}{K_i^{n_i} + x_1^{n_i}}\dfrac{K_{d\_inh}^{n_{d\_inh}}}{K_{d\_inh}^{n_{d\_inh}} + x_3^{n_{d\_inh}}} - x_4\Big], \end{cases} \tag{34}$$

with the Gaussian white noise as that in Eq. (2) of the main text. The diffusion matrix is set as $D(x_1, x_2, x_3, x_4) = I$. The variables x_1, x_2, x_3, x_4 denote concentration of Oct4, Sox2, mesendodermal, ectodermals separately.

We calculate from ODE the three stable fixed points $\mathbf{x}_1^* = (0.0218, 0.0218, 1, 0.0545)$, $\mathbf{x}_2^* = (0.0218, 0.0218, 0.0545, 1)$,

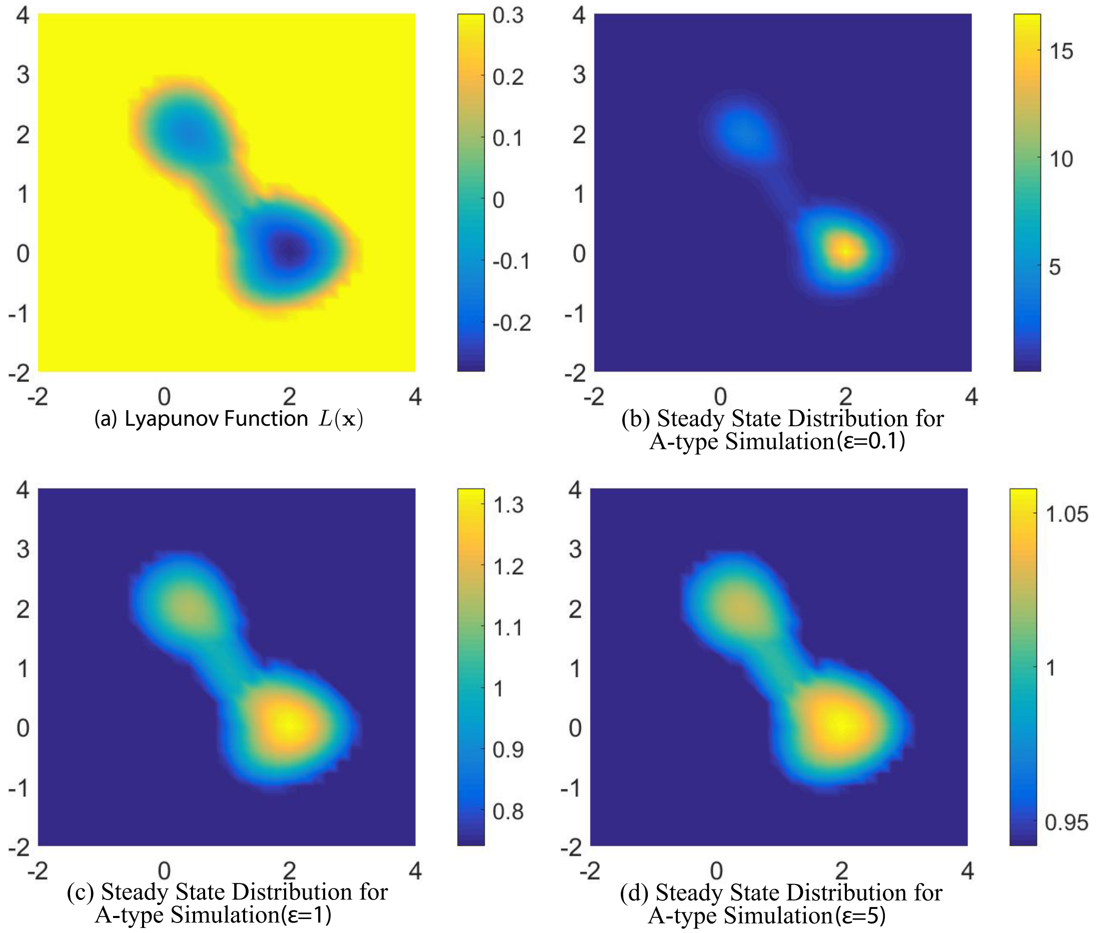

FIG. 2: (Color online) A-type integration preserves stability structure when noise increases. (a) Potential function by the Protocol II: we calculate the potential values of points in the x_1, x_2 mesh grid with steplength 0.2. When the calculated potential value is larger a chosen cutoff 0.3, it is set to be this cutoff value. Then, we add a constant to potential landscape such that the saddle point is the zero potential reference. The numerical parameters are $K = 500$, $T = 50$. (b,c,d) Steady state distribution by using $\rho_{ss}(\mathbf{x}) \propto \exp[-\phi(\mathbf{x})/\epsilon]$ (without normalization here) as A-type simulation under the noise strengths $\epsilon = 0.1, 1, 5$. The bistable dynamical structure is preserved when noise increases.

$\mathbf{x}_3^* = (1.3309, 1.3309, 0.4423, 0.4423)$ with the parameters given in [18], which represent three cell types correspondingly: the mesendodermal state, the ectodermal state, and the pluripotency state. We list in table III the height of potential barriers from between stable fixed points. The result shows that the differentiated mesendodermal and ectodermal states are more stable than the meta-stable state of pluripotent stem cell. This provides a understanding on why stem cells have a higher probability to transit toward more differentiated cells than the other way around. The result also allows us to predict the rate and timescale of transitions between cell types, which may lead to clinical methodologies to shepherd cells from one state into another.

VII. ODES FOR THE 38 DIMENSIONAL PROSTATE CANCER MODEL

We list below all ODEs for the 38 dimensional prostate cancer model [19]. We have conducted random parameter test, where Hill coefficients n_{ij} for each interaction between the i-th and j-th species ($i, j = 1, \cdots, 38$) are real numbers uniformly distributed in $[1, 30]$, and $K_{ij} = 2^{n_{ij}}$. The 50,000 tests showed that all stable states have more than 98-percent recurrence rate. Therefore, the computed stable states are insensitive to the detailed parameter setting, and are mainly determined by the structure of the regulation network. For clarity, we choose the same Hill coefficient n_{ij}

and K_{ij} for each equation below and let $n = 3$, $K = 10$.

$$\frac{dx_{pRb}}{dt} = \frac{K \cdot (x^n_{Cyclin\ D/Cdk4,6} + x^n_{Cyclin\ E/Cdk2})}{1 + K \cdot (x^n_{Cyclin\ D/Cdk4,6} + x^n_{Cyclin\ E/Cdk2})} - x_{pRb},$$

$$\frac{dx_{Cyclin\ D/Cdk4,6}}{dt} = \frac{K \cdot (x^n_{Myc} + x^n_{E2F})}{1 + K \cdot (x^n_{Myc} + x^n_{E2F})} - x_{Cyclin\ D/Cdk4,6},$$

$$\frac{dx_{Cyclin\ E/Cdk2}}{dt} = \frac{K \cdot (x^n_{Myc} + x^n_{E2F})}{1 + K \cdot (x^n_{Myc} + x^n_{E2F})} \times \frac{1}{1 + K \cdot (x^n_{p21} + x^n_{p27} + x^n_{PTEN})} - x_{Cyclin\ E/Cdk2},$$

$$\frac{dx_{Myc}}{dt} = \frac{K \cdot (x^n_{pRb} + x^n_{E2F} + x^n_{MAPK})}{1 + K \cdot (x^n_{pRb} + x^n_{E2F} + x^n_{MAPK})} \times \frac{1}{1 + K \cdot (x^n_{p53} + x^n_{TGF-\beta})} - x_{Myc},$$

$$\frac{dx_{E2F}}{dt} = \frac{K \cdot (x^n_{Myc} + x^n_{E2F})}{1 + K \cdot (x^n_{Myc} + x^n_{E2F})} \times \frac{1}{1 + K \cdot x^n_{p21}} - x_{E2F},$$

$$\frac{dx_{p21}}{dt} = \frac{K \cdot (x^n_{p53} + x^n_{AR} + x^n_{TNF-\alpha})}{1 + K \cdot (x^n_{p53} + x^n_{AR} + x^n_{TNF-\alpha})} \times \frac{1}{1 + K \cdot (x^n_{Myc} + x^n_{Akt})} - x_{p21},$$

$$\frac{dx_{p27}}{dt} = \frac{K \cdot (x^n_{PTEN} + x^n_{E-Cadherin})}{1 + K \cdot (x^n_{PTEN} + x^n_{E-Cadherin})} \times \frac{1}{1 + K \cdot (x^n_{Myc} + x^n_{Akt})} - x_{p27},$$

$$\frac{dx_{p53}}{dt} = \frac{K \cdot (x^n_{Myc} + x^n_{PTEN})}{1 + K \cdot (x^n_{Myc} + x^n_{PTEN})} \times \frac{1}{1 + K \cdot x^n_{Akt}} - x_{p53},$$

$$\frac{dx_{Caspase\ 3}}{dt} = \frac{K \cdot (x^n_{Cytochrome\ c} + x^n_{Caspase\ 8})}{1 + K \cdot (x^n_{Cytochrome\ c} + x^n_{Caspase\ 8})} \times \frac{1}{1 + K \cdot x^n_{XIAP}} - x_{Caspase\ 3},$$

$$\frac{dx_{Cytochrome\ c}}{dt} = \frac{K \cdot (x^n_{Caspase\ 3} + x^n_{Bad} + x^n_{Bax})}{1 + K \cdot (x^n_{Caspase\ 3} + x^n_{Bad} + x^n_{Bax})} \times \frac{1}{1 + K \cdot (x^n_{Bcl-2} + x^n_{Bcl-xL})} - x_{Cytochrome\ c},$$

$$\frac{dx_{Caspase\ 8}}{dt} = \frac{K \cdot (x^n_{TNF-\alpha} + x^n_{Fas})}{1 + K \cdot (x^n_{TNF-\alpha} + x^n_{Fas})} - x_{Caspase\ 8},$$

$$\frac{dx_{XIAP}}{dt} = \frac{K \cdot (x^n_{Akt} + x^n_{NF-\kappa B})}{1 + K \cdot (x^n_{Akt} + x^n_{NF-\kappa B})} \times \frac{1}{1 + K \cdot x^n_{Caspase\ 3}} - x_{XIAP},$$

$$\frac{dx_{Bcl-2}}{dt} = \frac{K \cdot (x^n_{VEGF} + x^n_{Integrin})}{1 + K \cdot (x^n_{VEGF} + x^n_{Integrin})} \times \frac{1}{1 + K \cdot (x^n_{p53} + x^n_{Caspase\ 3} + x^n_{TGF-\beta})} - x_{Bcl-2},$$

$$\frac{dx_{Bcl-xL}}{dt} = \frac{K \cdot (x^n_{EGF} + x^n_{IGF-1R})}{1 + K \cdot (x^n_{EGF} + x^n_{IGF-1R})} \times \frac{1}{1 + K \cdot x^n_{Caspase\ 3}} - x_{Bcl-xL},$$

$$\frac{dx_{Bim}}{dt} = \frac{1}{1 + K \cdot (x^n_{Akt} + x^n_{MAPK})} - x_{Bim},$$

$$\frac{dx_{Bad}}{dt} = \frac{1}{1 + K \cdot (x^n_{p21} + x^n_{Akt} + x^n_{MAPK})} - x_{Bad},$$

$$\frac{dx_{Bax}}{dt} = \frac{K \cdot (x^n_{p53} + x^n_{Bim})}{1 + K \cdot (x^n_{p53} + x^n_{Bim})} - x_{Bax},$$

$$\frac{dx_{Ras}}{dt} = \frac{K \cdot (x^n_{VEGF} + x^n_{AR} + x^n_{Integrin} + x^n_{IL-6})}{1 + K \cdot (x^n_{VEGF} + x^n_{AR} + x^n_{Integrin} + x^n_{IL-6})} \times \frac{1}{1 + K \cdot x^n_{p53}} - x_{Ras},$$

$$\frac{dx_{Akt}}{dt} = \frac{K \cdot (x^n_{AR} + x^n_{NF-\kappa B})}{1 + K \cdot (x^n_{AR} + x^n_{NF-\kappa B})} \times \frac{1}{1 + K \cdot x^n_{PTEN}} - x_{Akt},$$

$$\frac{dx_{PTEN}}{dt} = \frac{K \cdot x^n_{p53}}{1 + K \cdot x^n_{p53}} \times \frac{1}{1 + K \cdot x^n_{NF-\kappa B}} - x_{PTEN},$$

$$\frac{dx_{MAPK}}{dt} = \frac{K \cdot (x^n_{Ras} + x^n_{IGF-1R} + x^n_{Integrin} + x^n_{NF-\kappa B})}{1 + K \cdot (x^n_{Ras} + x^n_{IGF-1R} + x^n_{Integrin} + x^n_{NF-\kappa B})} \times \frac{1}{1 + K \cdot (x^n_{PTEN} + x^n_{MKP})} - x_{MAPK},$$

$$\frac{dx_{MKP}}{dt} = \frac{K\cdot(x_{MAPK}^n + x_{EGF}^n)}{1+K\cdot(x_{MAPK}^n + x_{EGF}^n)} - x_{MKP},$$

$$\frac{dx_{VEGF}}{dt} = \frac{K\cdot(x_{IL-6}^n + x_{COX-2}^n)}{1+K\cdot(x_{IL-6}^n + x_{COX-2}^n)} - x_{VEGF},$$

$$\frac{dx_{EGF}}{dt} = \frac{K\cdot(x_{AR}^n + x_{HIF}^n)}{1+K\cdot(x_{AR}^n + x_{HIF}^n)} - x_{EGF},$$

$$\frac{dx_{IGF-1R}}{dt} = \frac{K\cdot x_{AR}^n}{1+K\cdot x_{AR}^n} \times \frac{1}{1+K\cdot x_{p53}^n} - x_{IGF-1R},$$

$$\frac{dx_{AR}}{dt} = \frac{K\cdot(x_{EGF}^n + x_{IL-6}^n)}{1+K\cdot(x_{EGF}^n + x_{IL-6}^n)} \times \frac{1}{1+K\cdot x_{PTEN}^n} - x_{AR},$$

$$\frac{dx_{Integrin}}{dt} = \frac{K\cdot(x_{VEGF}^n + x_{EGF}^n + x_{TNF-\alpha}^n)}{1+K\cdot(x_{VEGF}^n + x_{EGF}^n + x_{TNF-\alpha}^n)} - x_{Integrin},$$

$$\frac{dx_{E-Cadherin}}{dt} = \frac{K\cdot x_{Akt}^n}{1+K\cdot x_{Akt}^n} \times \frac{1}{1+K\cdot(x_{TNF-\alpha}^n + x_{TGF-\beta}^n)} - x_{E-Cadherin},$$

$$\frac{dx_{HIF}}{dt} = \frac{K\cdot x_{Akt}^n}{1+K\cdot x_{Akt}^n} \times \frac{1}{1+K\cdot x_{p53}^n} - x_{HIF},$$

$$\frac{dx_{NF-\kappa B}}{dt} = \frac{K\cdot(x_{TNF-\alpha}^n + x_{IL-1}^n)}{1+K\cdot(x_{TNF-\alpha}^n + x_{IL-1}^n)} \times \frac{1}{1+K\cdot x_{i\kappa B}^n} - x_{NF-\kappa B},$$

$$\frac{dx_{i\kappa B}}{dt} = \frac{K\cdot x_{NF-\kappa B}^n}{1+K\cdot x_{NF-\kappa B}^n} \times \frac{1}{1+K\cdot(x_{Akt}^n + x_{EGF}^n + x_{TNF-\alpha}^n + x_{Fas}^n)} - x_{i\kappa B},$$

$$\frac{dx_{TNF-\alpha}}{dt} = \frac{K\cdot(x_{NF-\kappa B}^n + x_{IL-1}^n)}{1+K\cdot(x_{NF-\kappa B}^n + x_{IL-1}^n)} \times \frac{1}{1+K\cdot x_{IL-10}^n} - x_{TNF-\alpha},$$

$$\frac{dx_{IL-6}}{dt} = \frac{K\cdot x_{NF-\kappa B}^n}{1+K\cdot x_{NF-\kappa B}^n} \times \frac{1}{1+K\cdot(x_{p21}^n + x_{IL-10}^n)} - x_{IL-6},$$

$$\frac{dx_{IL-10}}{dt} = \frac{K\cdot(x_{TNF-\alpha}^n + x_{Fas}^n)}{1+K\cdot(x_{TNF-\alpha}^n + x_{Fas}^n)} \times \frac{1}{1+K\cdot x_{IL-10}^n} - x_{IL-10},$$

$$\frac{dx_{Fas}}{dt} = \frac{K\cdot x_{TNF-\alpha}^n}{1+K\cdot x_{TNF-\alpha}^n} \times \frac{1}{1+K\cdot x_{Ras}^n} - x_{Fas},$$

$$\frac{dx_{COX-2}}{dt} = \frac{K\cdot(x_{MAPK}^n + x_{NF-\kappa B}^n)}{1+K\cdot(x_{MAPK}^n + x_{NF-\kappa B}^n)} - x_{COX-2},$$

$$\frac{dx_{TGF-\beta}}{dt} = \frac{K\cdot x_{TNF-\alpha}^n}{1+K\cdot x_{TNF-\alpha}^n} - x_{TGF-\beta},$$

$$\frac{dx_{IL-1}}{dt} = \frac{K\cdot x_{NF-\kappa B}^n}{1+K\cdot x_{NF-\kappa B}^n} - x_{IL-1}.$$

[1] J. Shi, T. Chen, R. Yuan, B. Yuan, and P. Ao, *Relation of a new interpretation of stochastic differential equations to itô process,* J. Stat. Phys. **148**, 579 (2012).

[2] C. W. Gardiner, *Handbook of Stochastic Methods*, 3rd ed. (Springer-Verlag, Berlin, 2004).

[3] P. Lançon, G. Batrouni, L. Lobry, and N. Ostrowsky, *Drift without flux: Brownian walker with a space-dependent diffusion coefficient,* Europhys. Lett. **54**, 28 (2001).

[4] G. Volpe, L. Helden, T. Brettschneider, J. Wehr, and C. Bechinger, *Influence of noise on force measurements,* Phys. Rev. Lett. **104**, 170602 (2010).

[5] W. Horsthemke and R. Lefever, *Noise-Induced Transitions: Theory and Applications in Physics, Chemistry, and Biology*, 2nd ed. (Springer-Verlag, Berlin, 2006).

[6] F. Sagués, J. M. Sancho, and J. García-Ojalvo, *Spatiotemporal order out of noise,* Rev. Mod. Phys. **79**, 829 (2007).

[7] M. Assaf, E. Roberts, Z. Luthey-Schulten, and N. Goldenfeld, *Extrinsic noise driven phenotype switching in a self-regulating gene,* Phys. Rev. Lett. **111**, 058102 (2013).

[8] T. Biancalani, L. Dyson, and A. J. McKane, *Noise-induced bistable states and their mean switching time in foraging colonies,* Phys. Rev. Lett. **112**, 038101 (2014).
[9] Y. Tang, R. Yuan, and P. Ao, *Summing over trajectories of stochastic dynamics with multiplicative noise,* J. Chem. Phys. **141**, 044125 (2014).
[10] M. I. Freidlin and A. D. Wentzell, *Random Perturbations of Dynamical Systems*, 3rd ed. (Springer-Verlag, Berlin, 2012).
[11] R. Yuan and P. Ao, *Beyond itô versus stratonovich,* J. Stat. Mech. **2012**, P07010 (2012).
[12] H. A. Kramers, *Brownian motion in a field of force and the diffusion model of chemical reactions,* Physica **7**, 284 (1940).
[13] P. Hänggi, P. Talkner, and M. Borkovec, *Reaction-rate theory: fifty years after Kramers,* Rev. Mod. Phys. **62**, 251 (1990).
[14] C. Lv, X. Li, F. Li, and T. Li, *Constructing the energy landscape for genetic switching system driven by intrinsic noise,* PLoS one **9**, e88167 (2014).
[15] J. X. Zhou, M. Aliyu, E. Aurell, and S. Huang, *Quasi-potential landscape in complex multi-stable systems,* J. R. Soc. Interface **9**, 3539 (2012).
[16] D. K. Wells, W. L. Kath, and A. E. Motter, *Control of stochastic and induced switching in biophysical networks,* Phys. Rev. X **5**, 031036 (2015).
[17] T. S. Gardner, C. R. Cantor, and J. J. Collins, *Construction of a genetic toggle switch in escherichia coli,* Nature **403**, 339 (2000).
[18] J. Shu, C. Wu, Y. Wu, Z. Li, S. Shao, W. Zhao, X. Tang, H. Yang, L. Shen, X. Zuo, *et al.*, *Induction of pluripotency in mouse somatic cells with lineage specifiers,* Cell **153**, 963 (2013).
[19] X. Zhu, R. Yuan, L. Hood, and P. Ao, *Endogenous molecular-cellular hierarchical modeling of prostate carcinogenesis uncovers robust structure,* Prog. Biophys. Mol. Biol. **117**, 30 (2015).
